# The long non-coding RNA MEG8 induces an endothelial barrier through regulation of microRNA-370 and -494 processing

**DOI:** 10.1242/jcs.259671

**Published:** 2022-06-16

**Authors:** Veerle Kremer, Laura Stanicek, Eva van Ingen, Diewertje I. Bink, Sarah Hilderink, Anke J. Tijsen, Ilka Wittig, Lars Mägdefessel, Anne Yaël Nossent, Reinier A. Boon

**Affiliations:** 1 Amsterdam UMC location Vrije Universiteit, Department of Physiology, De Boelelaan 1117, 1081 HZ Amsterdam, The Netherlands; 2 Amsterdam Cardiovascular Sciences, Microcirculation, De Boelelaan 1108, 1081 HZ Amsterdam, the Netherlands; 3 Amsterdam Cardiovascular Sciences, Heart Failure & Arrhythmias, De Boelelaan 1108, 1081 HZ Amsterdam, the Netherlands; 4 Amsterdam UMC location University of Amsterdam, Department of Medical Chemistry, Meibergdreef 9, 1105 AZ Amsterdam, the Netherlands; 5 Department of Surgery and Einthoven Laboratory for Experimental Vascular Medicine, Leiden University Medical Center, Albinusdreef 2, 2333 ZA Leiden, the Netherlands; 6 Amsterdam UMC location University of Amsterdam, Department of Experimental Cardiology, Meibergdreef 9, 1105 AZ, Amsterdam, the Netherlands; 7 Functional Proteomics, SFB 815 Core Unit, Faculty of Medicine, Goethe University, 60590 Frankfurt am Main, Germany; 8 Department of Vascular and Endovascular Surgery, Klinikum rechts der Isar, Technical University of Munich, 81675 Munich, Germany; 9 Department of Medicine, Molecular Vascular Medicine Unit, Karolinska Institute, 17177 Stockholm, Sweden; 10 German Centre for Cardiovascular Research (DZHK), Partner site Munich Heart Alliance, Potsdamer Straße 58,10785 Berlin, Germany; 11 Departments of Laboratory Medicine and Internal Medicine II, Medical University of Vienna, 1090 Vienna, Austria; 12 Institute of Cardiovascular Regeneration, Goethe University, 60596 Frankfurt am Main, Germany; 13 German Centre for Cardiovascular Research (DZHK), Partner site Frankfurt Rhein/Main, Potsdamer Straße 58,10785 Berlin, Germany

**Keywords:** Aging, Endothelial barrier, Non-coding RNA, Post-transcriptional modification

## Abstract

The 14q32 locus is an imprinted region in the human genome which contains multiple non-coding RNAs. We investigated the role of the long non-coding RNA maternally expressed gene 8 (MEG8) in endothelial function and its underlying mechanism. A 5-fold increase in MEG8 was observed with increased passage number in human umbilical vein endothelial cells (HUVECs), suggesting MEG8 is induced during aging. MEG8 knockdown resulted in a 1.8-fold increase in senescence, suggesting MEG8 might be protective during aging. The endothelial barrier was also impaired after MEG8 silencing. MEG8 knockdown resulted in reduced expression of microRNA (miRNA)-370 and -494 but not -127, -487b and -410. Overexpression of miRNA-370 or -494 partially rescued the MEG8-silencing-induced barrier loss. Mechanistically, MEG8 regulates expression of miRNA-370 and -494 at the mature miRNA level through interaction with the RNA-binding proteins cold-inducible RNA-binding protein (CIRBP) and hydroxyacyl-CoA dehydrogenase trifunctional multi-enzyme complex subunit β (HADHB). Mature miRNA-370 and miRNA-494 were found to interact with CIRBP, whereas precursor miRNA-370 and miRNA-494 were found to interact with HADHB. Individual CIRBP and HADHB silencing resulted in downregulation of miRNA-370 and induction of miRNA-494. These results suggest MEG8 interacts with CIRBP and HADHB and contributes to miRNA processing at the post-transcriptional level.

## Abstract (precis)

**Summary:** MEG8 contributes to endothelial barrier integrity by regulating expression of miRNA-370 and miRNA-494, as well as processing of these miRNAs by interacting with RNA-binding proteins CIRBP and HADHB.

## INTRODUCTION

One of the essential functions of the endothelium is providing a semi-permeable barrier, allowing for paracellular and transcellular transport of liquids and solutes in a controlled manner. The endothelial barrier is regulated by protein complexes, such as adherens junctions and tight junctions. Adherens junctions connect the actin cytoskeleton of neighboring cells. Proteins in this complex include vascular endothelial (VE)-cadherin, α- and β-catenin (Krouwer et al., 2012; Pronk et al., 2019; López-Otín et al., 2013; Donato et al., 2015; Brandes et al., 2005). The capacity of endothelial cells (ECs) to divide is limited, and cellular senescence is suggested to occur *in vivo* during aging (Krouwer et al., 2012; Boisen et al., 2010). Signs of endothelial dysfunction are observed in senescent cells, including an impaired endothelial barrier and reduced endothelium-dependent vasodilation and angiogenesis. Furthermore, inflammation and apoptosis rates are elevated in senescent cells. In short, endothelial senescence contributes to structural and functional changes in the vasculature that can contribute to the progression of cardiovascular diseases (CVDs), such as atherosclerosis (Krouwer et al., 2012; Brandes et al., 2005; Cao et al., 2020; Jia et al., 2019).

Many different factors contribute to the regulation of the endothelium. The potential role for non-coding RNA in vascular regulation is rapidly emerging. Non-coding RNAs, as opposed to coding RNA, are transcribed but not translated into protein. Non-coding RNAs can be divided in short and long non-coding RNA (lncRNA) based on the size of the transcript (Michalik et al., 2014). LncRNAs are defined as transcripts longer than 200 nucleotides. Many lncRNAs share a common biogenesis pathway with mRNA. They are transcribed by RNA polymerase II, have a 5′-cap and are often spliced and poly-adenylated. In contrast to mRNA, lncRNA lacks an open reading frame (ORF). Often, their expression is more cell-specific than that of mRNAs, although their abundance is lower. LncRNAs can function *in cis* or *in trans*. Many lncRNAs are involved in processes such as chromatin remodelling, regulation of splicing and post-transcriptional modification. Alternatively, lncRNAs can act as scaffolds in protein complexes (Michalik et al., 2014; Mercer and Mattick, 2013; Mongelli et al., 2019; Marchese et al., 2017; Gupta et al., 2010). For example, the lncRNA ANRIL regulates miRNA-181a and Sirt1, promotes cell viability and prevents senescence of vascular smooth muscle cells (VSMCs). In addition, ANRIL prevents cell cycle arrest in aging VSMCs by inhibition of the p53–p21 pathway (Tan et al., 2019). The class of short non-coding RNAs includes microRNAs (miRNAs), with an average length of 22 nucleotides. Binding of a mature miRNA to the 3′-UTR of target mRNAs can induce mRNA degradation or inhibition of translation. One miRNA can bind multiple target transcripts and can therefore be involved in different cellular processes (O'Brien et al., 2018). The production of miRNAs is tightly controlled at multiple levels, during transcription as well as post-transcriptionally. miRNAs are transcribed into primary miRNAs (pri-miRNAs), which are processed into hairpin RNAs by the microprocessor complex, which includes DROSHA and DGCR8, to form precursor miRNA (pre-miRNA). This precursor is exported to the cytoplasm and further processed by DICER (also known as DICER1) to give rise to a miRNA duplex. One strand of the mature miRNA is predominantly incorporated in the RNA-induced silencing complex (RISC). Argonaute proteins, such as AGO2, are a crucial component of this complex and are able to bind single-strand miRNAs (Thum et al., 2008; O'Brien et al., 2018). Post-transcriptional processing of miRNAs also relies on RNA-binding proteins (RBPs). RBPs are essential regulators of cellular processes such as splicing and mRNA translation (Kim et al., 2017). An RBP can bind to either the stem or terminal loop of a miRNA transcript, and can enhance or inhibit post-transcriptional processing (Michlewski and Cáceres, 2019). For example, Lin-28 has been shown to bind the terminal loop of the let-7 g pri-miRNA and thereby block cleavage by the microprocessor complex (Viswanathan et al., 2008). LncRNAs are also known to bind RBPs to influence splicing and translation (Grammatikakis et al., 2014). For example, the lncRNA *gadd7* interacts with the RBP TDP-43 (also known as TARDBP) and regulates stability of *Cdk6* mRNA (Liu et al., 2012).

The 14q32 cluster is an imprinted genomic region that contains multiple non-coding RNAs. This cluster is well conserved in mammals and has been implicated in oncogenesis, proliferation and cell survival (Dimmeler and Ylä-Herttuala, 2014). Many miRNAs in this cluster have been implicated in vascular regulation. Interestingly, these miRNAs are predicted to regulate adherens junctions and extracellular matrix interactions, which are crucial in endothelial function (Dimmeler and Ylä-Herttuala, 2014; Welten et al., 2014; Benetatos et al., 2013). The 14q32 cluster also contains lncRNAs such as maternally expressed gene 3 (MEG3) and maternally expressed gene 8 (MEG8). MEG3 is among the most-studied transcripts of the region and is thought to be involved in angiogenesis, cell proliferation and differentiation (Benetatos et al., 2013; Boon et al., 2016). In a study by Zhang et al., MEG8 was found to be downregulated in a model of atherosclerosis in which VSMCs were treated with oxidized (ox)-LDL to simulate the high-lipid environment in atherosclerosis. Overexpression of MEG8 in this model was found to suppress cell proliferation and migration and induce apoptosis through the miRNA-181a/PPARα axis (Zhang et al., 2019). Also, Chen et al. described a potential protective role for MEG8 in the progression of liver disease. Silencing of MEG8 was shown to promote the activation of hepatic stellate cells and hepatocytes, and promote epithelial-to-mesenchymal transition through the Notch pathway, processes which are considered drivers of liver fibrosis (Chen et al., 2020).

This study reports on the role of MEG8 in regulation of the endothelium. MEG8 was upregulated during replicative senescence in endothelial cells. Silencing of MEG8 resulted in impaired endothelial barrier function, likely related to a reduction in miRNA-370 and -494 expression. Overexpression of these miRNAs partially restored the endothelial barrier. miRNA expression was found to be regulated by MEG8 at the post-transcriptional level. RBPs are thought to play a role in this. Cold-inducible RNA-binding protein (CIRBP) and hydroxyacyl-CoA dehydrogenase trifunctional multi-enzyme complex subunit β (HADHB) were identified as potential RBPs, and selected for further study.

## RESULTS

### MEG8 expression is increased in high passage HUVECs

**Fig. 1.**
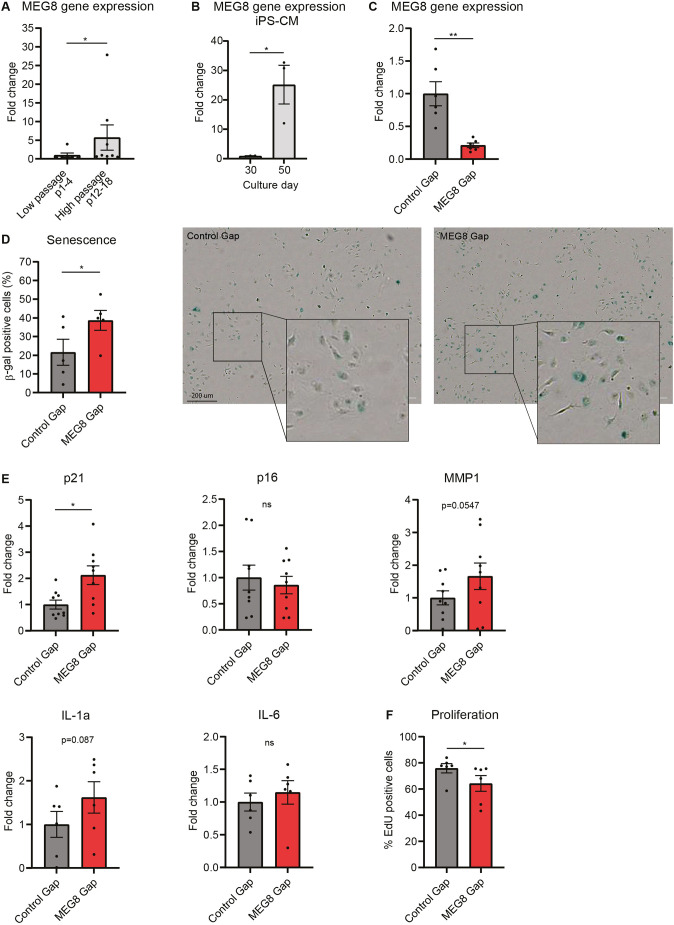
**MEG8 expression is induced in high passage endothelial cells.** (A) MEG8 mRNA levels were measured by RT-qPCR in HUVECs at low and high passage. Low passage (six samples) cells were between passage 1 and 4. High passage cells (eight samples) were between passage 12 and 18. Expression was normalized to RPLP0. Groups were compared using the Mann–Whitney test. (B) MEG8 mRNA levels were measured by RT-qPCR in iPSC-derived cardiomyocytes (iPS-CM). Cells cultured for 30 days were compared to 50 days in culture. Expression was normalized to RPLP0. 3 experiments were performed. Groups were compared using an unpaired two-tailed *t*-test. (C,D) HUVECs were transfected with MEG8 or control GapmeR (Gap) and (C) expression levels were measured 48 h after transfection by RT-qPCR. Expression is relative to RPLP0. Groups from six independent experiments were compared using a two-tailed paired *t*-test. (D) HUVECs were fixed 48 h after transfection stained using the senescence associated β-galactosidase staining kit. Cells were imaged 24 h after staining. Scale bar: 200 µm. Groups from five independent experiments were compared using a paired two-tailed *t*-test. (E) HUVECs were transfected with MEG8 or Control GapmeR and expression levels were measured 48 h after transfection by RT-qPCR. Expression is relative to RPLP0. Groups from 6–9 independent experiments were compared using a paired two-tailed *t*-test. (F) Proliferation was measured by EdU incorporation between 24–48 h after transfection. The percentage of proliferating cells is shown. Six experiments were performed. Groups were compared using a paired two-tailed *t*-test. Data are presented as mean±s.e.m. *P<0.05, **P<0.01, not significant (ns).

We investigated how 14q32 non-coding RNA contributes to CVDs. Aging is a well-known risk factor in many CVDs (North and Sinclair, 2012). To assess whether MEG8 is differentially expressed during aging *in vitro*, we measured MEG8 expression in human umbilical vein endothelial cells (HUVECs) at high and low passage number. A 5-fold increase in MEG8 expression was observed in high passage cells compared to low passage cells (Fig. 1A). In another model, a 25-fold increase in MEG8 expression was observed in human induced pluripotent stem cell (hiPSC)-derived cardiomyocytes at culture day 50 compared to at day 30 (Fig. 1B). To investigate the potential cellular mechanisms MEG8 is involved in, we silenced MEG8 by transfecting MEG8-targeting LNA-GapmeRs. Expression levels were reduced by 75% in MEG8 GapmeR-treated cells compared to those seen with a non-targeting control (Fig. 1C). As aging is linked to cellular dysfunction and senescence (North and Sinclair, 2012), we performed β-galactosidase staining, as a marker of senescence. Interestingly, a higher percentage of β-galactosidase-positive cells could be observed after MEG8 silencing (Fig. 1D). In addition, we assessed additional markers of senescence (as described by González-Gualda et al., 2021) and observed increased levels of mRNAs encoding p21 (encoded by *CDKN1A*) and a trend towards increased IL-1a and MMP1 expression. Other genes, such as p16 (*CDKN2A*) and IL-6, were not regulated (Fig. 1E). Proliferation was also reduced after loss of MEG8 (Fig. 1F). Taken together, HUVECs show several markers of senescence after MEG8 inhibition. We hypothesize that MEG8 is induced in high passage cells to prevent EC dysfunction, and loss of MEG8 exacerbates levels of markers of senescence.

### Loss of MEG8 leads to barrier disruption

**Fig. 2.**
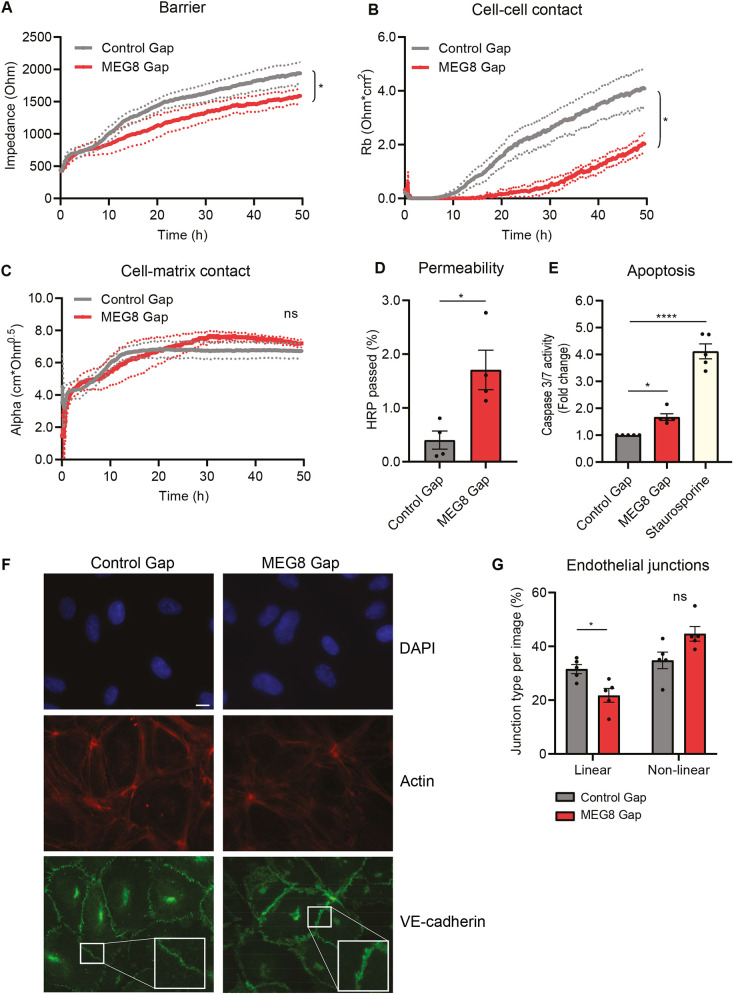
**MEG8 is required for maintaining the EC barrier and cell–cell junctions.** (A–C) HUVECs were seeded 24 h after transfection with MEG8 or control GapmeR (Gap) at a density of 100,000 cells per well in 8W10E ECIS plates. Impedance was measured continuously. By altering the frequency, the overall barrier (A), cell–cell contacts (B) and cell–matrix contacts (C) can be distinguished. The area under the curve was calculated for between 24 and 48 h. Groups were analyzed using a paired two-tailed *t*-test. Four independent experiments were performed. Continuous lines indicate the mean, dotted lines indicate s.e.m. (D) HUVECs were seeded on 3 µm filters 24 h after transfection. After 24 h, 5 µg/ml HRP was added to the top compartment. After 1 h, a sample was taken from the upper and lower compartments. HRP concentration was calculated by measuring absorbance. Data is presented as percentage of HRP in lower compartment. Four individual experiments were performed. Groups were analyzed using a paired two-tailed *t*-test. (E) HUVECs were seeded in a 96-well plate 45 h after transfection. Apoptosis was induced using 200 nm staurosporine. Caspase substrate was added and fluorescence was measured after 1 h. Fluorescence intensity was normalized to the control. Five individual experiments were performed. Groups were analyzed using one-way ANOVA with Dunnett test. (F,G) HUVECs were transfected, seeded on gelatin coated coverslips and grown to form junctions for 48 h. Cells were immunostained for VE-cadherin (green) and F-actin (red). Nuclei were stained with DAPI (blue). Scale bar: 10 µm. (G) Junction types were quantified from the images by overlaying a grid and scoring the most prevalent type junction in each square. Five independent experiments were performed, and two images per condition were scored in each experiment. Groups were compared using paired one-way ANOVA with Dunnett test. Data are presented as mean±s.e.m. *P<0.05, ****P<0.0001, not significant (ns).

To explore the functional effects of MEG8 in the endothelium, we assessed endothelial barrier after silencing of MEG8 using an electric cell–substrate impedance sensing (ECIS) setup. HUVECs were seeded on gold film coated electrodes and the electrochemical impedance was measured over time. By altering the frequency of the current, total barrier, cell–cell contacts and cell–matrix contacts can be distinguished. Total resistance was lower in MEG8-silenced cells, indicating an impaired barrier (Fig. 2A). More importantly, cell–cell contacts were also shown to be severely disrupted (Fig. 2B). In contrast, cell–matrix interactions were not severely affected (Fig. 2C). These findings were validated (Fig. S1A–D) using two additional GapmeRs, as well as a MEG8-targeting siRNA previously described by Chen et al. (2020). In addition to ECIS, a transwell assay was performed in which HUVECs were seeded on a filter and horseradish peroxidase (HRP) passage is measured as an indication of macromolecular permeation. In accordance with the ECIS results, permeability was increased after loss of MEG8 (Fig. 2D). Apoptosis was measured to determine whether barrier impairment could be due to an excessive loss of cells. A 2-fold increase in caspase 3 and 7 activity was detected after loss of MEG8, suggesting that cells indeed undergo apoptosis (Fig. 2E). However, the induction of apoptosis was not substantially different to that in control cells, and we hypothesize additional cellular changes underlie barrier loss. Therefore, we investigated the possibility of altered VE-cadherin expression underlying the loss of barrier, since this is an important component of cell–cell junctions (Duong and Vestweber, 2020). There was no change in VE-cadherin mRNA levels (Fig. S1E). There was a reduction in total VE-cadherin protein after silencing of MEG8, although this difference was small at ∼15% (Fig. S1F). To analyze the different types of cellular junctions, immunofluorescence staining was performed (Fig. 2F,G). Linear junctions, which are suggestive of a more stable and quiescent barrier (Malinova and Huveneers, 2018), were observed more in control cells compared to upon MEG8 knockdown (Fig. 2G). Non-linear junctions, such as reticular junctions and focal adherens junctions, which were observed more often after loss of MEG8, are considered disrupted junctions and are an indication of a more unstable barrier (Malinova and Huveneers, 2018). We also investigated the effect of MEG8 loss during aging. The EC barrier was measured in HUVECs at low and high passage using ECIS. Endothelial barrier was impaired in high passage cells compared to low passage cells. Loss of MEG8 resulted in further barrier impairment in high passage cells, although this was not statistically significant (Fig. S1G–J). In conclusion, loss of MEG8 leads to endothelial barrier impairment and disruption of cell–cell junctions but not cell–matrix junctions.

### MEG8 interacts with CIRBP and HADHB and regulates expression of miRNA-370 and miRNA-494

**Fig. 3.**
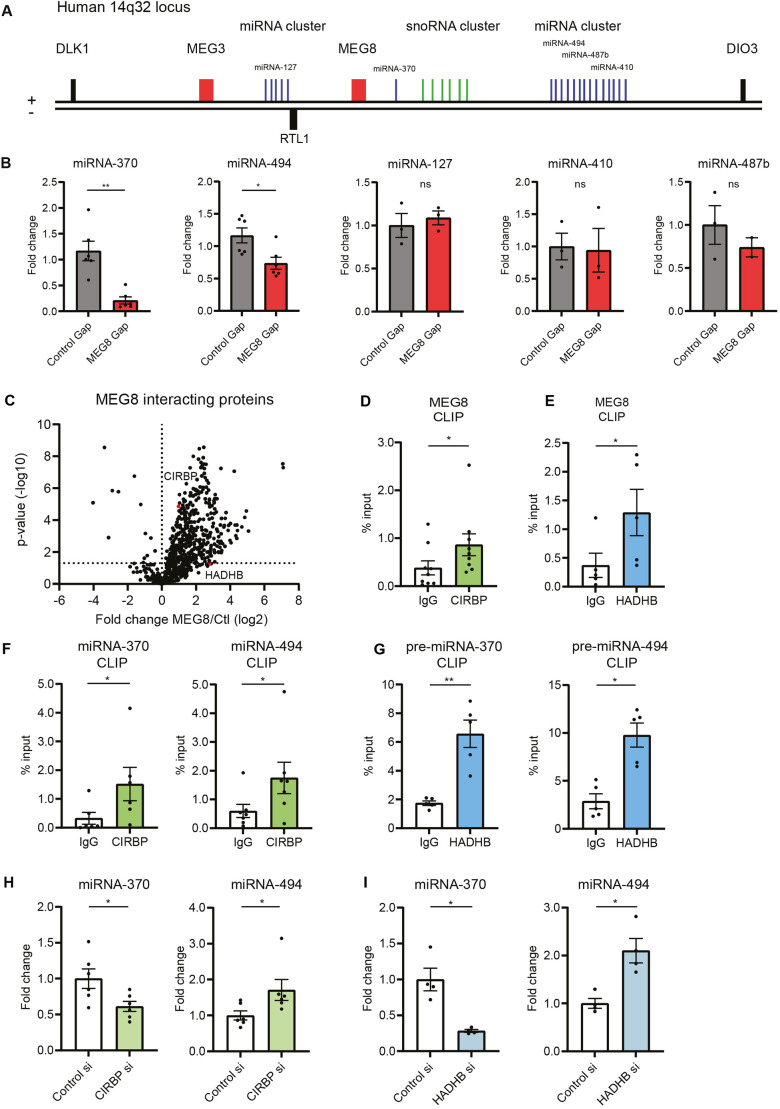
**MEG8 interacts with CIRBP and HADHB, and regulates expression of miRNA-370 and -494.** (A) Schematic overview of the human 14q32 locus. (B) HUVECs were transfected with MEG8 or control GapmeR (Gap) and mature miRNA expression levels were measured 48 h after transfection by RT-qPCR. Expression is relative to miRNA-191. Three to six individual experiments were performed. Groups were compared using a paired two-tailed *t*-test. (C) Elutions from MEG8-antisense purification were analyzed by mass spectrometry. A volcano plot is shown depicting proteins significantly enriched by an anti-MEG8 oligonucleotide compared to a non-targeting control. CIRBP and HADHB are highlighted in red. Six individual experiments were performed. (D,E) MEG8 binding to CIRBP (D) and HADHB (E) was analyzed in HUVECs by RT-qPCR following CLIP. Non-targeting IgG was used as a control. Enrichment was quantified relative to input. Five to nine individual experiments were performed. Data were analyzed by a paired two-tailed *t*-test. (F,G) miRNA and pre-miRNA binding to CIRBP (F) and HADHB (G) was analyzed in HUVECs by RT-qPCR following CLIP. Primers for pre-miRNA levels also detect pri-miRNA levels. Pri-miRNA primers are specific for pri-miRNA levels. Non-targeting IgG was used as a control. Enrichment was quantified relative to input. Five to seven individual experiments were performed. Data were analyzed by a paired two-tailed *t*-test. (H) HUVECs were transfected with CIRBP siRNA or control siRNA and expression levels were measured 48 h after transfection by RT-qPCR. Expression is relative to miRNA-191. Six individual experiments were performed. Groups were compared using a paired two-tailed *t*-test. (I) HUVECs were transfected with HADHB siRNA or control siRNA and expression levels were measured 48 h after transfection by RT-qPCR. Expression is relative to miRNA-191. Four individual experiments were performed. Groups were compared using a paired two-tailed *t*-test. Data are presented as mean±s.e.m. *P<0.05, **P<0.01, not significant (ns).

MEG8 is located on the imprinted 14q32 cluster, consisting of different lncRNAs, snoRNAs and miRNAs (Benetatos et al., 2013). We asked whether loss of MEG8 also affects expression of miRNAs in the same cluster. A subset of miRNAs was selected and expression levels were measured by reverse transcription quantitative PCR (RT-qPCR). These miRNAs were selected because they were distributed along the locus and several of these miRNAs have already been shown to play a role in vascular remodeling (Welten et al., 2014). We observed a reduction in the mature miRNA levels of miRNA-370-3p and -494-3p but no change in that of miRNA-127-3p, -487b-3p or -410-3p expression (Fig. 3A,B; Fig. S2E). These results suggest MEG8 regulates expression of specific miRNAs, but not the miRNA cluster as a whole. To assess whether change in mature miRNA expression is due to a transcriptional effect, we measured expression levels of pri-miRNA and pre-miRNA. There was no significant difference in pri- or pre-miRNA expression levels between control and MEG8-silenced cells (Fig. S2A–D). Of note, RT-qPCR primers for pre-miRNAs also detect pri-miRNAs, whereas pri-miRNA primers are specific to pri-miRNA. Since we observed no change in pri-miRNA levels, we concluded that any changes measured by pre-miRNA primers are indeed changes in pre-miRNA levels. These findings suggest MEG8 regulates miRNA-370 and -494 at a post-transcriptional level rather than by regulating transcription itself. We further investigated several crucial steps in miRNA processing. In order to be processed by DICER, pre-miRNAs need to be exported to the cytoplasm. Cell fractionation showed no change in pre-miRNA export from the nucleus to the cytoplasm (Fig. S2F). By using cross-linking immunoprecipitation (CLIP), miRNA association with AGO2 was quantified. There was no change in miRNA association with AGO2 after loss of MEG8 (Fig. S2G). Alternatively, miRNA stability could be altered following MEG8 silencing. HUVECs were treated with the RNA polymerase inhibitor actinomycin D, after which miRNA levels were measured (Fig. S2H). Generally, miRNAs have a slow rate of turnover, and therefore cells were treated for 24 h before collecting RNA (Guo et al., 2015). Levels of miRNA-191, which is believed to be stably expressed, are not changed compared to DMSO control (Peltier and Latham, 2008). Loss of MEG8 did not affect stability of miRNA-370 or -494. Alternatively, miRNA processing can be post-transcriptionally regulated by RBPs (Michlewski and Cáceres, 2019). Different RBPs interact specifically with distinct pre-miRNAs (Treiber et al., 2017). To clarify the role of MEG8 in miRNA processing, potential protein interaction partners for MEG8 were identified by RNA antisense purification and mass spectrometry. Prior to RNA antisense purification, MEG8 accessibility was examined by RNase H digestion and oligonucleotide binding was subsequently validated by RT-qPCR (Fig. S3A). Probe 4 was used to design the antisense purification probe. RNA antisense purification and subsequent mass spectrometry showed no MEG8 interaction with miRNA-processing proteins, such as DROSHA, DGCR8 or DICER. Instead, CIRBP and HADHB were shown to interact with MEG8 (Fig. 3C; Table S2), and were selected for further analysis since they had previously been described to be involved in processing of other 14q32 miRNAs (Downie Ruiz Velasco et al., 2019). The interaction of MEG8 with CIRBP and HADHB was confirmed by CLIP (Fig. 3D,E; Fig. S3B,C). In addition, CIRBP was found to interact with mature miRNAs whereas HADHB interacted with pre-miRNAs (Fig. 3F,G; Fig. S3D,E). To investigate the role of CIRBP and HADHB in miRNA processing, both were silenced using siRNAs. Subsequent RT-qPCR showed no change in pre-miRNA levels (Fig. S3F,G). Both CIRBP and HADHB silencing resulted in a downregulation of miRNA-370 and an upregulation of miRNA-494 (Fig. 3H,I). These results suggest CIRBP and HADHB play a role in miRNA-370 and -494 processing at the post-transcriptional level.

### miRNA overexpression following MEG8 knockdown contributes to a stable endothelial barrier

**Fig. 4.**
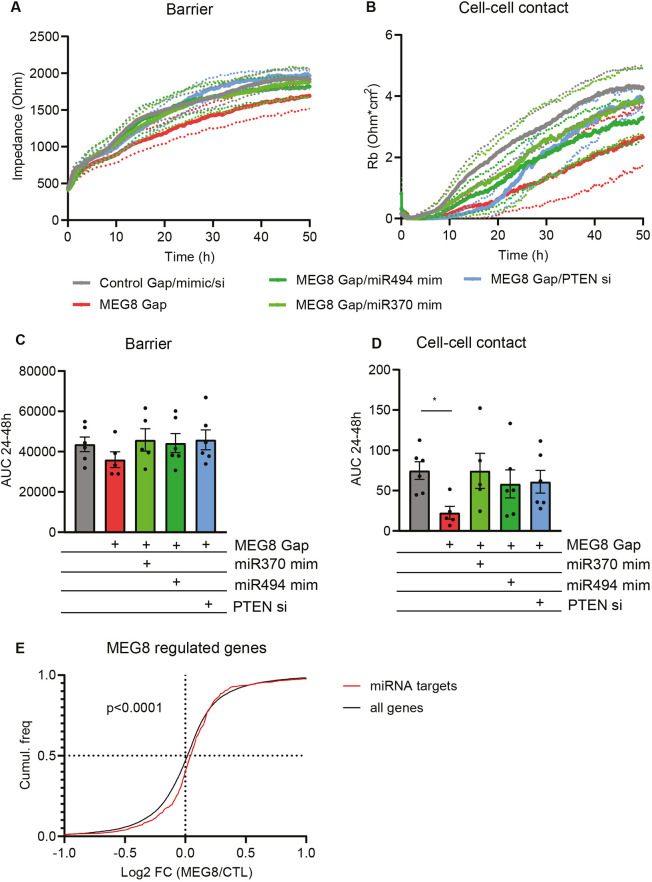
**Overexpression of miRNA-370 or -494 restores the EC barrier.** (A,B) HUVECs transfected as described in Fig. 2A–C and seeded at 24 h after transfection at a density of 100,000 cells per well in 8W10E ECIS plates. Impedance was measured continuously. By altering the frequency, the overall barrier (A) and cell–cell contacts (B) can be distinguished over time. Continuous lines indicate the mean, dotted lines indicate s.e.m. (C,D) The area under the curve of experiments as in A and B was quantified between 24 h and 48 h, and groups were analyzed using a paired one-way ANOVA with Dunnett test. Six individual experiments were performed. (E) Gene expression in control and MEG8 GapmeR 1-treated HUVECs was obtained from previously published data (GSE186616; Kremer et al., 2022). miRNA-370 and miR-494 targets were predicted using online available tools (miRWalk, miRanda, RNA22 and TargetScan). The fold change of miRNA targets was compared to expression of all genes comparing MEG8 knockdown and control conditions. Groups were compared using a Kolmogorov–Smirnov test. Data are presented as mean±s.e.m. *P<0.05.

Since loss of MEG8 resulted in a decrease in miRNA expression, we aimed to determine whether overexpression of miRNA-370 and -494 could rescue the loss of barrier observed after loss of MEG8. miRNAs were overexpressed using miRNA mimics, double-stranded oligonucleotides that mimic the function of endogenous mature miRNA. The functional effect of miRNA overexpression was assessed by ECIS. Silencing of MEG8 resulted in barrier disruption, whereas additional overexpression of either miRNA-370 or -494 partially restored the barrier and also improved cell–cell contact (Fig. 4A–D). In addition, inhibition of miRNA-370 and -494 also resulted in an impaired endothelial barrier (Fig. S4A,B). We selected PTEN as a potential downstream target of miRNA-370 and -494. RNAhybrid (Krüger and Rehmsmeier, 2006; https://bibiserv.cebitec.uni-bielefeld.de/rnahybrid) predicted miRNA binding to PTEN. Furthermore, it has been observed that S1P2R-Rho–ROCK–PTEN signaling plays a role in the disruption of adherens junctions and the induction of paracellular permeability (Sanchez et al., 2007). We assessed PTEN protein levels by western blotting after MEG8 knockdown and miRNA overexpression (Fig. S4C). We observed a slight increase in PTEN after MEG8 knockdown, which was abrogated by miRNA-494 overexpression. Functionally, inhibition of PTEN restored EC barrier function after loss of MEG8 (Fig. 4A–D). Since miRNAs have multiple target genes, we further assessed the effect of MEG8 on predicted miRNA-370 and miRNA-494 targets, and selected genes that are predicted to be targeted by both miRNA-370 and -494. The following databases were used: miRWalk, miRanda, RNA22 and TargetScan. Genes that were predicted to be a target in at least three of the databases were selected. We obtained mRNA expression levels after MEG8 knockdown from our previously published dataset (GSE186616; Kremer et al., 2022). When comparing the fold change of predicted miRNA targets to all genes regulated by MEG8, we observed a shift towards increased expression of miRNA targets after loss of MEG8 (Fig. 4E). This would suggest that loss of MEG8 and subsequent reduction in miRNA expression results in a global derepression of miRNA-370 and -494 target genes. Taken together, these results suggest MEG8 contributes to endothelial barrier maintenance through regulation of specific miRNA expression.

## DISCUSSION

Here, we show that the lncRNA MEG8 is regulated during endothelial aging. Loss of MEG8 was accompanied by an impaired endothelial barrier, more specifically a loss of cell–cell contacts. Mechanistically, MEG8 regulates expression of miRNA-370 and -494 at the post-transcriptional level through interaction with the RBPs CIRBP and HADHB.

Aging is a known risk factor in vascular disease, and it has been shown that there is impairment of cellular function during aging (Bianchessi et al., 2015). LncRNAs are known to regulate many cellular processes, and we aimed to further elucidate the role of lncRNAs in regulating the endothelial function. The lncRNA SENCR has previously been described to maintain EC barrier integrity through stabilization of VE-cadherin at the membrane (Lyu et al., 2019). MEG8 expression is induced during replicative senescence in HUVECs. In a study by Casella et al., a 2-fold induction in MEG8 expression was observed in human aortic endothelial cells (HAECs) after irradiation-induced senescence (Casella et al., 2019). In a study of aging in mice, multiple non-coding RNAs were found to be upregulated during aging, many of them from the 12F1 cluster, the mouse homolog of 14q32. *Meg8*, also known as *Rian* in mice, was found to be upregulated in murine liver during aging (White et al., 2015). Silencing of MEG8 results in increased senescence-associated β-galactosidase in HUVECs. Interestingly, overexpression of MEG8 has been shown to repress miRNA-34a in human cancer cells (Terashima et al., 2018). miRNA34a is induced in senescent HUVECs, and miRNA-34a overexpression has been shown to inhibit proliferation and induce senescence (Ito et al., 2010). Silencing of MEG8 also contributes to further barrier loss in high passage endothelial cells. We hypothesize that MEG8 is a protective molecule in the endothelium that is induced upon aging to prevent further endothelial dysfunction. A protective function for MEG8 in the liver has previously reported by Chen et al. (2020). To confirm whether MEG8 acts to prevent endothelial dysfunction in aging, an important next step would be to overexpress MEG8 in ECs. This would potentially show the protective effects of MEG8 in the endothelium, also at high passage. There was a small decrease in VE-cadherin protein following MEG8 knockdown. VE-cadherin is among the most important proteins in endothelial junctions (Duong and Vestweber, 2020). We observed no change in VE-cadherin mRNA levels, suggesting a mechanism besides transcriptional regulation by MEG8. Also, we found no evidence of MEG8 interacting with VE-cadherin protein. Therefore, it is possible that the reduction in VE-cadherin protein is secondary to barrier disruption.

The 14q32 cluster is a large polycistronic miRNA cluster consisting of 54 miRNAs. It has been shown that miRNAs in this cluster are differentially regulated during ischemic disease in mice. Interestingly, expression patterns vary between different miRNAs, with some being upregulated early in this process and others being unaffected (Downie Ruiz Velasco et al., 2019). Upon MEG8 silencing in HUVECs, we observed a reduction in mature miRNA-370 and -494 expression levels, but not other miRNAs from the same cluster. There is likely no direct transcriptional regulation by MEG8, since there was no change in miRNA precursor levels. We observed no change in pre-miRNA export or miRNA stability. Alternatively, it is possible that lncRNAs act as a miRNA sponge (Mongelli et al., 2019). We observed no increase in miRNA expression upon MEG8 knockdown, therefore this hypothesis appears unlikely. MEG8 was shown to interact with RBPs CIRBP and HADHB, which have been previously implicated in the processing of 14q32 miRNAs (Downie Ruiz Velasco et al., 2019). CIRBP is a stress responsive gene that is upregulated during mild hypothermia and under hypoxic (1% O_2_) conditions. CIRBP can bind the 3′-UTR of multiple genes, stabilizing the mRNA and thereby promoting translation. CIRBP shares amino acid sequence similarity with RNA-binding motif protein 3 (RBM3) in its RNA-binding domain as well as in a conserved RNA recognition motif region. RBM3 is thought to contribute to miRNA biogenesis by promoting association to DICER. To our knowledge, the role of CIRBP in miRNA processing is currently not fully understood (Zhu et al., 2016; Chang et al., 2016; Pilotte et al., 2011). HADHB is part of the mitochondrial trifunctional protein complex, which catalyzes long-chain fatty acid oxidation. Several metabolic enzymes, such as glutamate dehydrogenase, have been shown to bind RNA. HADHB binds the 3′-UTR of REN mRNA and destabilizes the transcript (Adams et al., 2003; Hollams et al., 2002; Dagher et al., 2021). Currently, the exact mechanism through which HADHB contributes to miRNA processing is not known. In our model, CIRBP binds to mature miRNA-370 and miRNA-494, whereas HADHB binds to precursor miRNA-370 and miRNA-494, although the exact binding site is not known. miRNA-370 was found to be downregulated following CIRBP or HADHB knockdown; therefore, we hypothesize that CIRBP and/or HADHB induce processing of miRNA-370. MEG8 recruits CIRBP and HADHB to miRNA-370 to enhance its processing. On the other hand, mature miRNA-494 expression was upregulated after CIRBP or HADHB silencing. This would suggest that CIRBP and HADHB inhibit miRNA-494 processing. miRNA-494 levels were downregulated after MEG8 silencing, and we hypothesize that MEG8 prevents binding of CIRBP and/or HADHB to miRNA-494, which allows processing to take place. An RBP that acts as a negative regulator of processing of one miRNA, but a positive regulator of another has been described previously. Binding of RBP hnRNPA1 results in a change in conformation of the stem of pri-miRNA-18a, which increases accessibility of DROSHA cleavage sites (Michlewski et al., 2008). A negative regulatory function has been described for hnRNPA1 as well, with hnRNPA1 binding the terminal loop of pri-let-7a and blocking DROSHA-mediated processing (Michlewski and Cáceres, 2010). The two miRNAs affected by hnRNPA1 are substantially different in terms of sequence and structure. miRNA-18a is much less thermodynamically stable than let-7a. Variation in both structure and sequence of miRNAs are thought to determine the result of RBP binding (Michlewski and Cáceres, 2010), and it is likely that CIRBP, HADHB and MEG8 regulate processing of miRNA-370 and miRNA-494 in a similar manner. Future experiments could determine whether MEG8 regulates other miRNAs as well. For example, overexpression of MEG8 has been shown to induce transcriptional repression of miRNA-34a and miRNA-203 in human lung cancer cells (Terashima et al., 2018).

miRNA-370 or -494 overexpression after loss of MEG8 partially rescued endothelial barrier function. Overexpression of miRNA-370 has been found to inhibit angiogenic activity in HUVECs and dermal microvascular ECs (HDMECs) and reduce proliferation (Gu et al., 2019). miRNA-494 has been previously shown to target both pro- and anti-apoptotic genes in cardiomyocytes, which points to a more complex role for miRNA-494 in tissue homeostasis (Wang et al., 2010). Inhibition of miRNA-494 was shown to prevent migration, invasion and proliferation in gliomas through the PTEN/Akt pathway (Li et al., 2015). In addition to miRNA-494, miRNA-370 was shown to target PTEN in gastric cancer and inhibit proliferation (Zeng et al., 2016). We investigated PTEN as a potential downstream target of the miRNA, and indeed silencing of PTEN did improve EC barrier function. This is in accordance with findings by Sanchez et al. (2007). On the other hand, deletion of *Pten* in the mouse brain has been found to increase the transcellular permeability of the blood–brain barrier (Cui et al., 2021). These findings suggest that the role of PTEN could be dependent on vascular bed or cell treatment. We did not observe a complete restoration of barrier function after miRNA overexpression. However, it should be noted that up to 60% of mammalian mRNAs can be bound by miRNAs (Friedman et al., 2009), therefore it is likely that multiple mRNAs are targeted that positively or negatively impact the endothelial barrier. Additionally, it is possible that MEG8 is targeting several molecular pathways at the same time, and that other pathways might also contribute to barrier maintenance. This is further strengthened by the fact that miRNA inhibition did result in barrier impairment, but this was not as pronounced as that seen upon knockdown of MEG8. Finally, another important point to consider in this study is the finding that antisense oligonucleotides, such as GapmeRs, can contribute to premature RNA polymerase II transcription termination (Lai et al., 2020; Lee and Mendell, 2020). In addition to RNA degradation, there could also be additional effects on MEG8 transcription induced by the GapmeR. In summary, this study demonstrates the role of the 14q32 locus in vascular barrier dysfunction, and could contribute to future therapies to prevent endothelial dysfunction and CVD progression.

## MATERIALS AND METHODS

### Cell culture

Human umbilical vein endothelial cells (HUVECs) were purchased from Lonza (CC-2519, lots p1028 and p1032) and cultured in endothelial cell medium (ECM) supplemented with 1% endothelial cell growth supplement (ECGS), 1% penicillin/streptomycin (P/S) and 5% FBS (all Sciencell). HUVECs were used between passage 1 and 4 for experiments. Cells at passage 12 to 18 were used as a model for replicative senescence. To inhibit transcription, cells were treated with actinomycin D (5 µg/ml, Sigma-Aldrich) in DMSO for 24 h. Cells were cultured at 37°C and 5% CO_2_ and cell numbers were determined using the Countess II Automated Cell Counter (Thermo Fisher Scientific). Culture medium was regularly tested for mycoplasma contamination.

### Human iPSC culture

The fully characterized hiPSC line is derived from dermal fibroblasts of a healthy male as previously published (Shinnawi et al., 2015). Informed consent was obtained after approval by the IRB committee of Amsterdam Medical Center. Colonies of hiPSCs were cultured in mTeSR-1 (StemCell Technologies) on plates coated with growth factor-reduced Matrigel (1:200 dilution, Corning). Cells were collected using 0.5 mM EDTA (Invitrogen), passaged every 4–6 days, and replated in mTeSR-1 supplemented with 2 μM thiazovivin (Selleck Chemicals). mTeSR-1 medium was replaced daily, except for the first day after passaging.

### Cardiac differentiation of hiPSC

Cardiac differentiation was performed using the following protocol with adaptations (Burridge et al., 2014). Differentiation was induced 3–5 days after passaging by changing to CDM3 medium (RPMI-1640; Gibco) with 500 μg/ml human serum albumin (Sigma-Aldrich), 213 μg/ml L-ascorbic acid 2-phosphate (Sigma-Aldrich) and 1% penicillin/streptomycin, supplemented with 6 μM CHIR99021 (Stemgent) for 2 days, followed by CDM3 with 2 μM Wnt-C59 (Selleck Chemicals) for 2 days. From day 4 to day 10, medium was changed every other day to RPMI/B27 medium [RPMI-1640; 2% B27 supplement minus insulin (Gibco) and 1% penicillin/streptomycin]. Spontaneous contraction could be identified from day 8 onwards. Starting at day 10, medium for the hiPSC cardiomyocytes (hiPSC-CMs) was replaced with CDM3 medium without glucose [RPMI-1640 without glucose (Gibco), 20 mM sodium lactate (Sigma-Aldrich) dissolved in 1 M HEPES solution] and changed once a week for at least 2 weeks to metabolically select the cardiomyocytes (Tohyama et al., 2013). After selection, medium was replaced once a week with CDM3 medium with glucose. Purified populations of cardiomyocytes were harvested at day 30 and 50 after start of differentiation by washing in PBS (Gibco) and addition of 1 ml TriReagent (Sigma-Aldrich) to the well. RNA was isolated according to the manufacturer's protocol (Direct-zol RNA miniprep kit, Zymo).

### Transfection

HUVECs were transfected at 60–80% confluence with 50 nM locked nucleic acid (LNA) GapmeR (Exiqon) or siRNAs (Sigma-Aldrich) using Lipofectamine RNAiMax (Thermo Fisher Scientific) in OptiMEM Glutamax (Gibco). As a control, non-targeting LNA GapmeR or siRNA was transfected. The medium was replaced with full ECM after 4 h. For miRNA overexpression, miRNA mimics (Dharmacon) were used at a final concentration of 50 nM and transfected using the same protocol. Sequences and catalog numbers can be found in Table S1.

### RNA isolation and RT-qPCR

Total RNA was isolated using TRIzol (Thermo Fisher Scientific) and the Direct-zol RNA miniprep kit (Zymo Research) according to the manufacturer's instructions. For RT-qPCR, 1000 ng of total RNA was reverse transcribed using oligo(dT) and random primers (iScript cDNA synthesis kit, Bio-Rad) according to the manufacturer's instructions. RT-qPCR was undertaken using the iQ SYBR Green Supermix (Bio-Rad) in a CFX96 or CFX384 Touch Real-Time PCR Detection System (Bio-Rad). Gene expression analysis was done using the 2^−ΔCt^ method. For mature miRNA quantification, RNA was reverse transcribed using the Taqman microRNA Reverse Transcription kit (Thermo Fisher Scientific) and quantified using miRNA-specific Taqman qPCR kits (Thermo Fisher Scientific). The following miRNAs were analyzed: hsa-mir-hsa-127, hsa-mir-370, hsa-mir-410, hsa-mir-487b, hsa-miR-494 and hsa-mir-191. Expression was normalized to the levels of miRNA-191 and gene expression analysis was done using the 2^−ΔCt^ method. All sequences can be found in Table S1.

### Senescence-associated β-galactosidase staining

Senescence-associated β-galactosidase activity as analyzed using the senescence associated β-galactosidase staining kit (Cell Signaling Technologies #9860). Cells were transfected as described above and fixed 48 h after transfection. After 24 h incubation, staining solution was removed and cells were covered with 70% glycerol. Images were taken using the Zeiss DIM LED microscope. The number of positive cells and total cell number was counted using ImageJ.

### Proliferation

Cell proliferation was measured using a 5-ethynyl-2′-deoxyuridine (EdU) incorporation assay (Click-iT EdU cell proliferation kit, Thermo Fisher Scientific) according to the manufacturer's instructions. Cells were seeded in eight-well µ-slides (Ibidi) at a density of 30,000 cells per well. EdU at a final concentration 10 µM was added to each well and fixation and staining was undertaken after 24 h. Cells were imaged using the Axio Observer Z1.0 microscope (Zeiss). Total cell number and the number of EdU-positive cells were counted.

### Western blotting

HUVECs were lysed 48 h after transfection in radioimmunoprecipitation assay (RIPA) buffer (Sigma-Aldrich) with protease and phosphatase inhibitors (Halt inhibitor cocktail, Thermo Fisher Scientific). Lysates were centrifuged at 20,000 ***g*** for 10 min and protein concentration was measured using Pierce BCA protein assay kit (Thermo Fisher Scientific). Equal amounts (10 µg) of protein were separated on SDS-PAGE gels and blotted on nitrocellulose membranes. Membranes were blocked in block buffer (TBS with 1% Tween 20, 5% BSA and 0.05% sodium azide) and incubated overnight with primary antibodies diluted in block buffer. GAPDH or β-actin were used as loading controls. Secondary antibodies (Dako) were incubated for 2 h. Bands were visualized using enhanced chemiluminescence (ECL, Amersham, GE Healthcare) on the AI600 (Amersham, GE Healthcare). Band intensity was quantified using ImageQuantTL. Full-size images can be found in Fig. S5. Antibodies and dilutions can be found in Table S1.

### Immunofluorescent staining

Cells were seeded on coverslips coated with 1% gelatin and grown until confluent in 48 h. Cells were washed in PBS++ (PBS supplemented with 0.5 mM MgCl_2_ and 1 mM CaCl_2_), fixed in 4% paraformaldelyde for 10 min and permeabilized in 0.1% Triton X-100 for 10 min at room temperature. Blocking was done in 2% BSA in PBS++ for 1 h at room temperature. Cells were incubated with primary antibody in blocking buffer overnight at 4°C. After washing with PBS++, coverslips were incubated with secondary antibody in blocking buffer for 1 h at room temperature. Coverslips were mounted with Mowiol (Sigma Aldrich). Microscopy was performed on the Axio Observer Z1.0 microscope (Zeiss). Junctions were quantified by overlaying a grid and scoring the dominant type of junction in each square. Antibodies and dilutions can be found in Table S1.

### Endothelial barrier function assay

Endothelial barrier was measured using electrical cell–substrate impedance sensing (ECIS, Applied Biophysics) at the multi-frequency setting. Prior to seeding cells, electrodes of the 8W10E plate (Ibidi) were coated with 10 mM l-cysteine (Sigma Aldrich) and 1% gelatin (Merck). Cells were seeded at a density of 100,000 cells per well in full ECM.

### Transwell assay

Macromolecular permeability was measured by passage of horseradish peroxidase (HRP) through a monolayer of HUVECs. Thin-certs (3 µm, Greiner Bio-one) were coated with 1% gelatin and 5 µg/ml fibronectin (Roche) and cells were seeded at a density of 100,000 cells per well in ECM. When a stable barrier had formed, medium in the upper compartment was replaced with 150 µl ECM containing 5 µg/ml HRP (Roche). After 1 h, a sample was taken from the upper and lower compartments. HRP concentration was calculated by measuring absorbance after adding TMB (Upstate/Milliore) and sulfuric acid (1 M) to stop the reaction. Absorbance was read at 450 nm using the Epoch plate reader (BioTek). Data is presented as percentage of HRP in lower compartment.

### Proliferation assay

Cell proliferation was measured using a 5-ethynyl-2′-deoxyuridine (EdU) incorporation assay (Click-iT EdU cell proliferation kit, Thermo Fisher Scientific). HUVECs were seeded 24 h after transfection in eight-well µ-slides (Ibidi) before addition of 10 µM EdU. Fixation and staining was done after 24 h EdU incubation. Cells were imaged using the ZOE fluorescence cell imager (Bio-Rad). Total cell number and EdU-positive cells were counted.

### Apoptosis

Caspase-3 and -7 activity was measured as a marker of apoptosis using the Apo ONE Homogenous Caspase-3/7 assay (Promega). Briefly, HUVECs were seeded in a 96-well plate 45 h after transfection and left to recover for 3 h. Staurosporine (Sigma Aldrich) was added to control cells at a final concentration of 200 nM to induce apoptosis. After 4 h incubation, caspase reagent was diluted in buffer and added to each well. Complete ECM was used as a blank. Fluorescence was measured at excitation/emission 485/521 nm using the FLUOstar Galaxy (BMG).

### Cellular fractionation

One 15 cm dish of HUVECs was used per condition. Cells were transfected with MEG8 or Control GapmeR and RNA was isolated after 48 h. Cells were washed in cold PBS and collected by scraping and centrifugation (5 min, 500 ***g***, 4°C). The pellet was resuspended in 200 µl buffer A (10 mM Tris-HCl pH 7.5, 10 mM NaCl, 3 mM MgCl_2_, 0.5% NP-40), incubated on ice for 5 min and centrifuged (3 min, 1000 ***g***, 4°C). TRIzol was added to the supernatant (cytoplasmic fraction) and the pellet was washed twice in buffer A. The pellet was resuspended in buffer B (10 mM Tris-HCl pH 7.5, 150 mM NaCl, 3 mM MgCl_2_), incubated on ice for 5 min and centrifuged (3 min, 1000 ***g***, 4°C). The pellet (nuclear fraction) was resuspended in TRIzol and RNA was isolated from each fraction. Pre-miRNA levels were measured using RT-qPCR. Sequences can be found in Table S1.

### RNase H accessibility assay

DNA oligonucleotides targeting the RNA of interest were designed using the Stellaris Designer Tool and used to analyze the accessibility of multiple sections of the RNA. One confluent 15 cm dish of HUVECs was washed in cold PBS, and cells were collected by scraping followed by another wash step. The pellet was resuspended in 100 µl lysis buffer [50 mM Tris-HCl pH 8, 150 mM NaCl, 0.5% NP-40, 1× Halt protease inhibitor cocktail (Thermo Fisher Scientific)], incubated for 30 min on ice and cleared by centrifugation (10 min, 20,000 ***g***, 4°C). Supernatant was adjusted to a volume of 1 ml with buffer (50 mM Tris-HCl pH 8, 60 mM NaCl, 75 mM KCl, 3 mM MgCl_2_, 10 mM DTT, 40 U RNase inhibitor (Thermo Fisher Scientific)]. 100 μl lysate was incubated with 100 pmol DNA oligonucleotide for 2 h at 4°C under rotation. 2.5 U RNase H (NEB) was added and incubated for 20 min at 37°C and 350 rpm. TRIzol was added and regions of the RNA were analyzed by RT-qPCR. Primers were designed to amplify a region of ∼150 nucleotides around the potentially bound DNA oligonucleotide. Sequences can be found in Table S1.

### RNA antisense purification and mass spectrometry

LncRNA–protein complexes were analyzed using affinity pulldown. Briefly, 150 µl streptavidin C1 beads (Thermo Fisher Scientific) per reaction were washed three times in wash buffer (50 mM Tris-HCl pH 8, 150 mM NaCl, 0.05% NP-40). Beads were blocked using 300 µg yeast RNA and 150 µg glycogen in 1 ml wash buffer. Beads were incubated for 2 h at 4°C under rotation. One confluent 15 cm dish of HUVECs was used per reaction. Cells were washed in PBS and collected using a scraper, followed by another wash step. Lysis was done in buffer L [50 mM HCl pH 8, 150 nM NaCl, 1 mM EDTA, 1% NP-40, 1× Halt protease inhibitor cocktail (Thermo Fisher Scientific)] on ice for 30 min followed by centrifugation (20,000 ***g***, 10 min, 4°C). Supernatant was adjusted to a volume of 1.1 ml (50 mM Tris-HCl pH 8, 150 mM NaCl, 75 mM KCl, 3 mM MgCl_2_, 10 mM DTT and 80 U RNase inhibitor). 100 µl was kept as input and 1 ml lysate was pre-cleared for 2 h at 4°C with 50 μl pre-blocked streptavidin C1 beads. Supernatant was separated from the beads and desthiobiotin-modified 2′O-Me-RNA anti-MEG8 or control oligonucleotide (200 pmol, IDT) was added to 1 ml pre-cleared lysate and incubated for 1 h at 37°C under rotation. Oligonucleotide sequences can be found in Table S1. RNA–oligonucleotide complexes were captured using 100 μl pre-blocked and washed beads for 1 h at 37°C after which the supernatant was separated from the beads. TRIzol was added to supernatant fraction to check MEG8 enrichment. Beads were washed twice in mild wash buffer (20 mM Tris-HCl pH 8, 10 mM NaCl, 1 mM EDTA and 0.05% NP-40) and once in mild wash buffer without NP-40. Elution was performed using 10 mM d-biotin (Invitrogen) in water for 30 min at 37°C under rotation. 1/8th of the eluate was treated with TRIzol to determine enrichment via RT-qPCR; 7/8th of the lysate was snap frozen in liquid nitrogen and used for mass spectrometry analysis. Mass spectrometry was performed using the Q Exactive Plus (Thermo Fisher Scientific) with an Dionex Ultimate 3000 ultra-high performance liquid chromatography unit (Thermo Fisher Scientific) and Nanospray Flex Ion-Source (Thermo Fisher Scientific). Data analysis was performed in MaxQuant 1.5.3.30 and Perseus 1.5.6.0. Sequences can be found in Table S1. Full analysis can be found in Table S2.

### Cross-linking RNA immunoprecipitation

For CLIP, 1–5 µg antibody was coupled to 50 µl Dynabeads protein G beads (10003D, Thermo Fisher Scientific) overnight at 4°C under rotation. One confluent 15 cm dish of HUVECs was used per condition. HUVECs were crosslinked with 50 mJ/cm^2^ UV light, washed in PBS and lysed with lysis buffer [50 mM Tris-HCl pH 8, 150 mM NaCl, 1 mM EDTA, 0.5% NP-40 and Halt protease inhibitor cocktail (Thermo Fisher Scientific)]. The supernatant was cleared by centrifugation (10,000 ***g*** for 10 min at 4°C) and the supernatant was diluted in 1 ml lysis buffer without NP-40. Protein G beads were washed three times in binding buffer (50 mM Tris-HCl pH 8, 150 mM NaCl, 1 mM EDTA and 0.05% NP-40) and lysate was incubated with the beads for 4 h at 4°C under rotation. Beads were washed in binding buffer and treated with 6.4 U proteinase K (NEB P8107S) per condition in proteinase K buffer (200 mM Tris-HCl pH 8, 25 mM EDTA, 300 mM NaCl and 2% SDS) for 30 min at 50°C. An equal volume of phenol/chloroform/isomylalcohol (Roth) was added for RNA extraction. Phase separation was done by centrifugation (20,000 ***g***, 10 min). Then 2.5× volume ethanol was added and RNA was precipitated overnight at −20°C. Washing was performed using 70% ethanol and the pellet was resuspended in MilliQ. RNA enrichment was measured by RT-qPCR. Mature miRNA enrichment was measured by using the Taqman miRNA kit. Alternatively, after incubation of beads with the lysate, beads were washed, resuspended in 5× sample buffer (312.5 mM Tris-HCl pH 6.8, 50% glycerol, 0.37 mM Bromophenol Blue, 10% SDS and 2.5% β-mercaptoethanol) and analyzed by western blotting as described above. To avoid detection of the IgG heavy and light chain, VeriBlot detection reagent (Abcam) was used to detect protein bands. Primers, antibodies and concentration can be found in Table S1.

### Statistical analysis

Data is presented as mean±s.e.m. GraphPad Prism 9 was used for the analysis. When comparing two groups, a paired or unpaired two-tailed *t*-test or Mann–Whitney test was performed. When comparing more than two groups, one-way analysis of variance (ANOVA) was performed including Holm–Sidak correction for multiple testing. Since knockdown efficiency varied per experiment, we performed paired analysis when comparing each treatment with its own control, within individual experiments. *P*<0.05 was considered significant.

## Supplementary Material

Supplementary information

Reviewer comments
