# Supplementary material for: The long non-coding RNA MEG8 induces an endothelial barrier through regulation of microRNA-370 and -494 processing
Source: J Cell Sci. 2022 Jun 16;135(12):jcs259671. doi: 10.1242/jcs.259671 (PMC9270956; doi:10.1242/jcs.259671)
Supplement: Supplementary information [file joces-135-259671-s1.pdf]

Supplemental figure 1

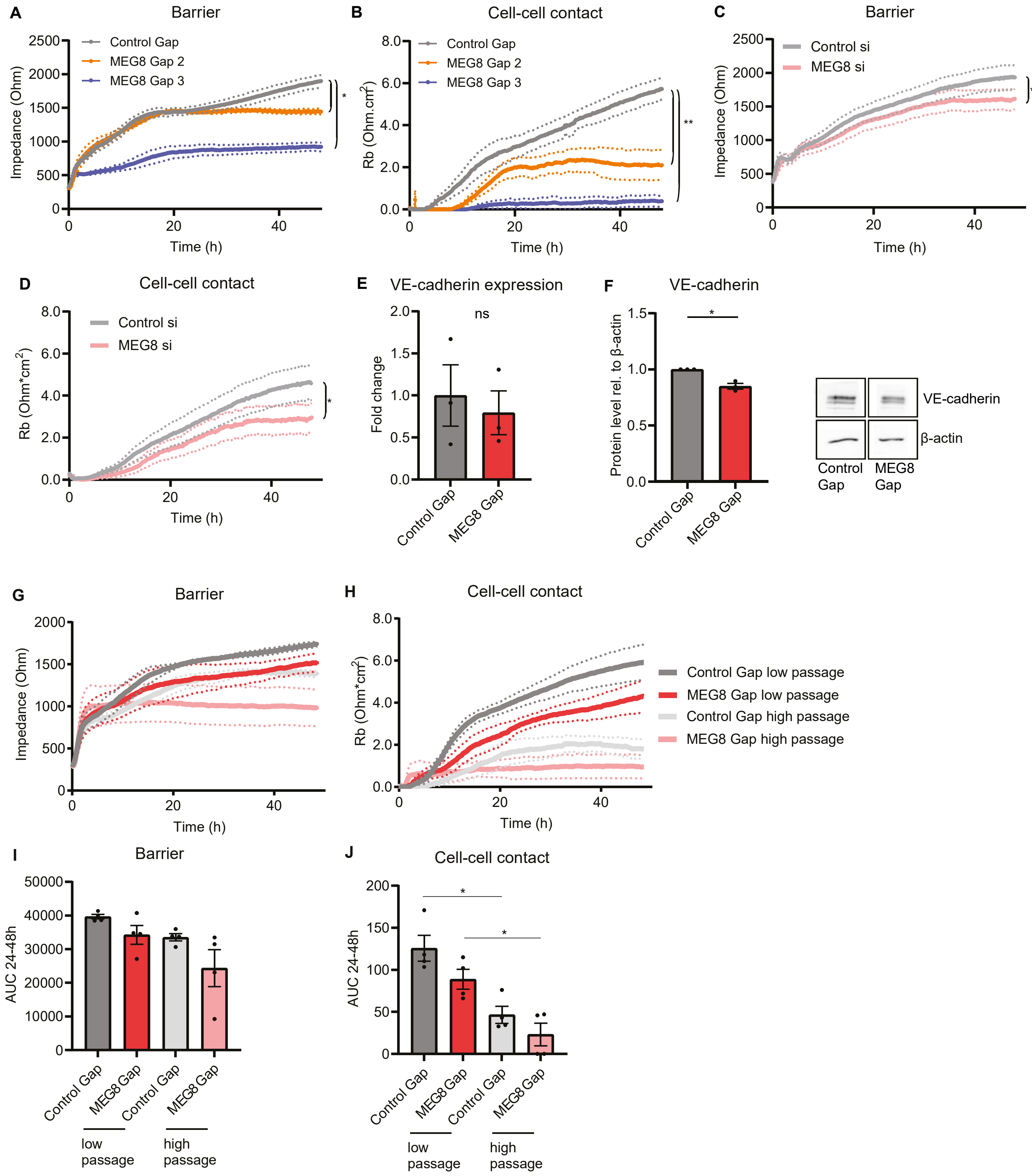

Journal of Cell Science • Supplementary information

Fig. S1. Loss of barrier after knockdown of MEG8. A-D: HUVECs were transfected with MEG8 GapmeR 2 and 3 or siRNA 1. Cells were seeded 24 hours after transfection at a density of 30 000 cells per well in 96W10idf ECIS plates. Impedance was measured continuously. By altering the frequency, overall (**A,C**) barrier and (**B,D**) cell-cell contact can be distinguished. Area under the curve was calculated between 24 and 48 hours. For **A-B**, 4 independent experiments were performed. For **C-D**, 7 independent experiments were performed. Groups were analysed using ANOVA. Continuous lines indicate the mean, dotted lines indicate SEM. E-**F**: HUVECs were transfected with MEG8 or control GapmeR and **E**: VE-cadherin expression levels were measured 48 hours after transfection by RT-qPCR. Expression is relative to RPLP0. 3 independent experiments were performed. Groups were compared using paired t-test. **F**: Relative levels of VE-cadherin were determined by Western Blotting. Images were cropped for clarity. Band intensities were determined using ImageQuant TL and normalized to β-actin. Groups were compared using paired t-test. **G-H**: HUVECs at high and low passage were seeded 24 hours after transfection at a density of 40 000 cells per well in 96W10idf ECIS plates. Impedance was measured continuously. (**I-J**) Area under the curve for (**G**) and (**H**) was calculated between 24 and 48 hours. 4 independent experiments were performed. Groups were analysed using unpaired ANOVA. Data are presented as mean ± SEM. Significance was indicated as: * $p < 0.05$, ** $p < 0.01$, *** $p < 0.001$, not significant (ns).

Journal of Cell Science • Supplementary information

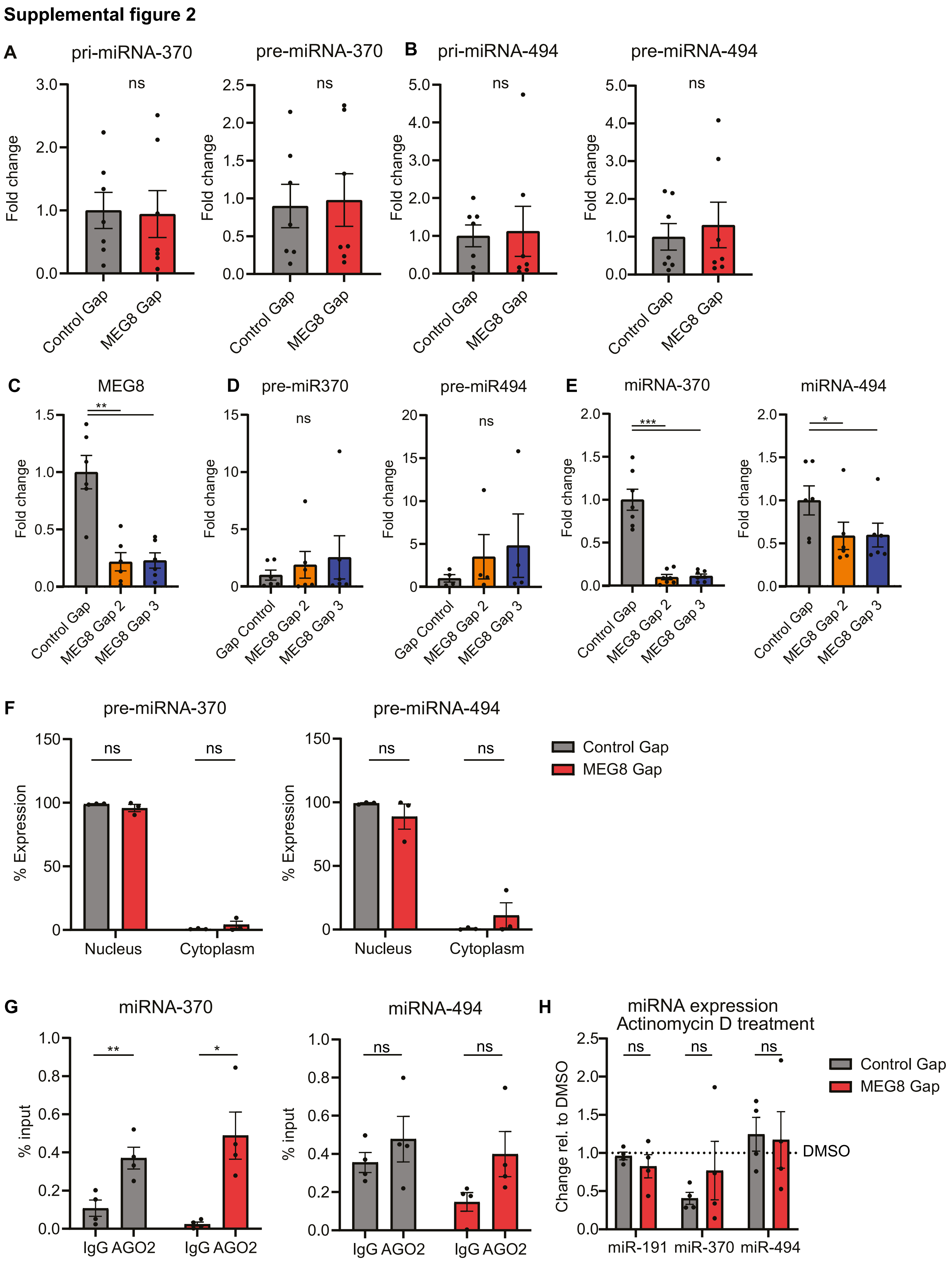

Journal of Cell Science • Supplementary information

Fig. S2. A-B: miRNA expression is regulated at the posttranscriptional level. HUVECs were transfected with MEG8 or control GapmeR and primary (**A**) and precursor (**B**) miRNA levels were measured 48 hours after transfection by RT-qPCR. Primers for pre-miRNA levels also detect pri-miRNA levels. Pri-miRNA primers are specific for pri-miRNA levels. Expression was relative to U6. 7 independent experiments were performed. Groups were analysed using paired t-test. **C-E**: HUVECs were transfected with MEG8 GapmeR 2 and 3 or Control GapmeR and RNA was collected after 48 hours. RNA expression was measured by RT-qPCR. **C**: MEG8 expression was measured relative to RPLP0. **D**: Precursor miRNA expression was measured relative to U6. **E**: Mature miRNA expression was measured relative to miRNA-191. 4-6 experiments were performed. Groups were compared using paired ANOVA. **F**: Nuclear/cytoplasmic fractions were separated 48 hours after transfection. Cellular distribution of pre-miRNA-370 and pre-miRNA-494 was analysed by RT-qPCR. 3 experiments were performed. Groups were analysed using one way ANOVA. **G**: miRNA binding to AGO2 was analysed in HUVECs by RT-qPCR following CLIP. Non-targeting IgG was used as a control. Enrichment was quantified relative to input. 3 experiments were performed. Groups were analysed using one way ANOVA. **H**: HUVECs were treated with actinomycin D or DMSO for 24 hours prior to RNA isolation. miRNA expression was measured using RT-qPCR. Fold change relative to DMSO treatment is shown for each condition. 4 experiments were performed. Data are presented as mean ± SEM. Significance was indicated as: * $p < 0.05$, ** $p < 0.01$, *** $p < 0.001$, not significant (ns).

Journal of Cell Science • Supplementary information

Supplemental figure 3

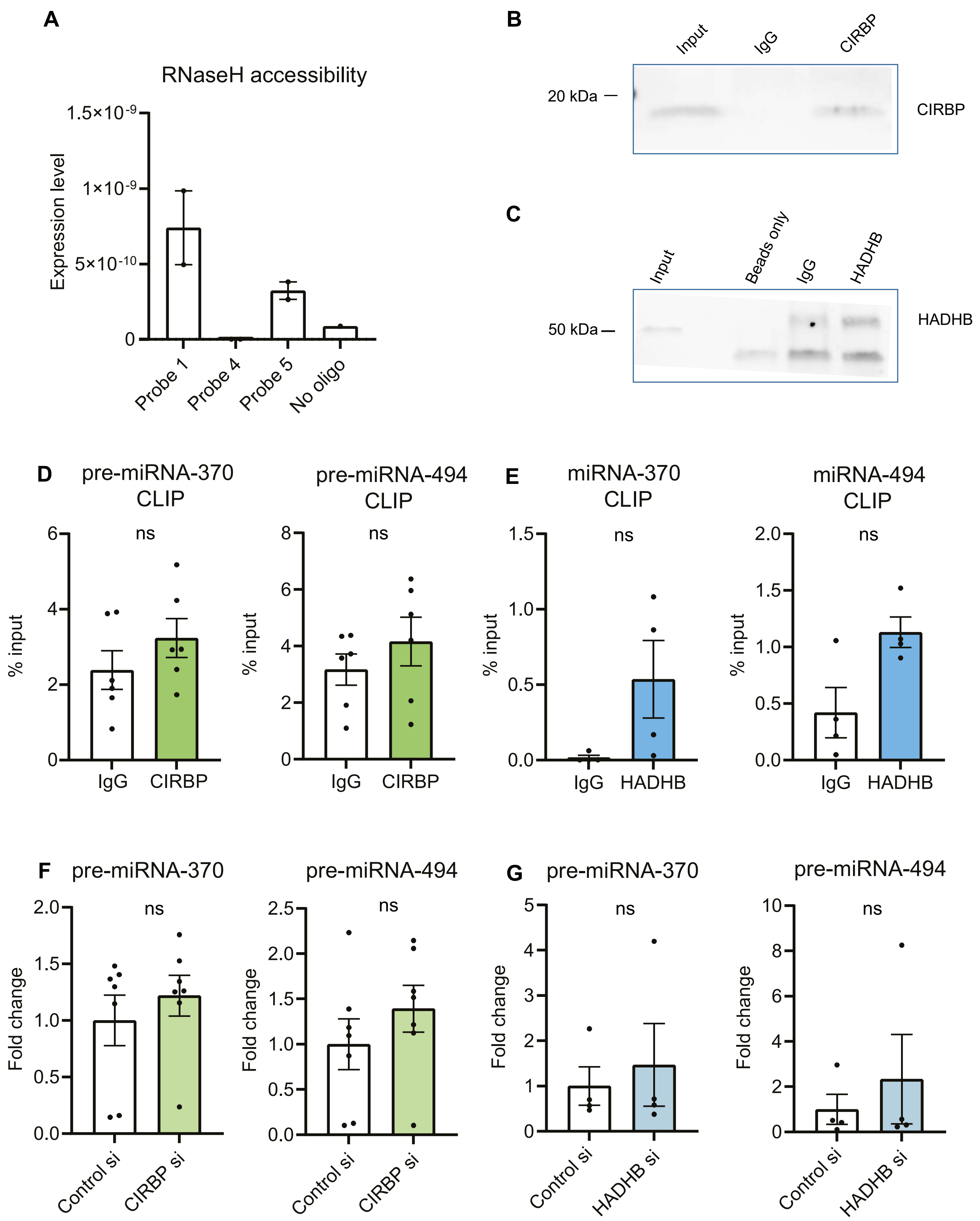

Journal of Cell Science • Supplementary information

Fig. S3. miRNA interaction with CIRBP and HADHB. A: DNA oligonucleotides targeting the RNA of interest were designed to analyse the accessibility of multiple sections of the RNA. Cell lysate was incubated with DNA oligonucleotide and treated with RNase H. Primers were designed to amplify by RT-qPCR a region of approximately 150 nucleotides around the potentially bound DNA oligonucleotide. 2 experiments were performed. **B**: CIRBP enrichment was assessed by Western blot following CLIP. Input is approximately 2% of the total lysate. The predicted size of CIRBP is 19 kDa. **C**: HADHB enrichment was assessed by Western blot following CLIP. Input is approximately 2% of the total lysate. The predicted size of HADHB is 50 kDa. **D**: pre-miRNA binding to CIRBP was analysed in HUVECs by RT-qPCR following CLIP. Primers for pre-miRNA levels also detect pri-miRNA levels. Pri-miRNA primers are specific for pri-miRNA levels. Non-targeting IgG was used as a control. Enrichment was quantified relative to input. 6 experiments were performed. **E**: Mature miRNA binding to HADHB was analysed in HUVECs by RT-qPCR following CLIP. Non-targeting IgG was used as a control. Enrichment was quantified relative to input. 4 experiments were performed. **F**: HUVECs were transfected with CIRBP siRNA or control siRNA and pre-miRNA expression levels were measured 48 hours after transfection by RT-qPCR. Expression is relative to U6. 7 experiments were performed. Groups were compared using paired t-test. **G**: HUVECs were transfected with HADHB siRNA or control siRNA and expression levels were measured 48 hours after transfection by RT-qPCR. Pre-miRNA expression is relative to U6. 4 experiments were performed. Groups were compared using paired t-test. Data are presented as mean ± SEM. Significance was indicated as: * $p < 0.05$, **$p < 0.01$, ***$p < 0.001$, not significant (ns).

Journal of Cell Science • Supplementary information

Supplemental figure 4

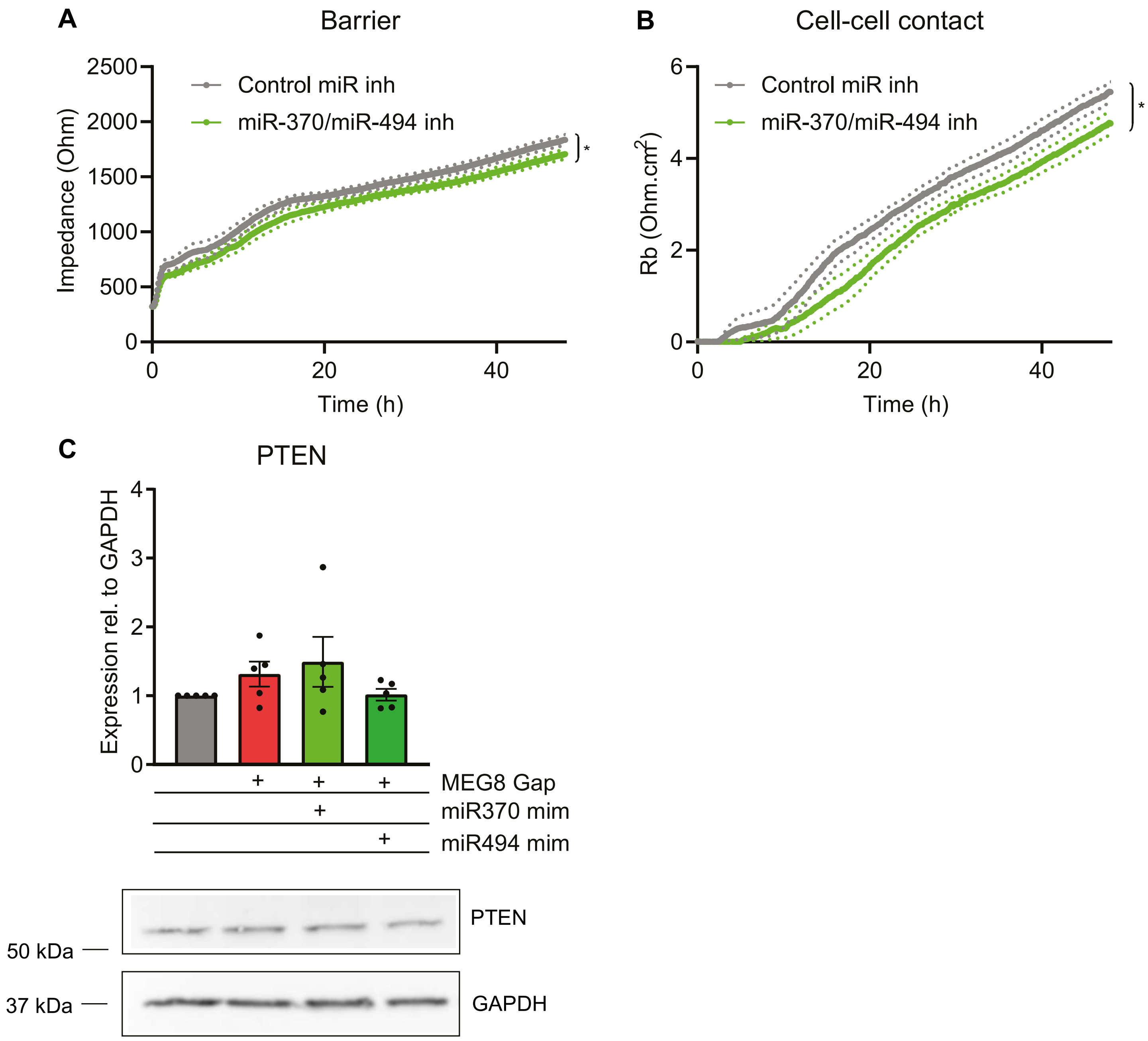

Fig. S4. Endothelial barrier impairment after miRNA inhibition. A-B: HUVECs transfected and seeded 24 hours after transfection at a density of 30 000 cells per well in 96W10E ECIS plates. Impedance was measured continuously. Continuous lines indicate the mean, dotted lines indicate SEM. The area under the curve of (**A**) and (**B**) was quantified between 24 and 48 hours and groups were analysed using paired t-test. In total, 7 experiments were performed. **C**: PTEN protein levels were determined using Western blot. Cell lysates were collected 48 hours after transfection. GAPDH was used as a loading control. Images were cropped for clarity. Band intensity was quantified using ImageQuant. 5 experiments were performed. Groups were compared using ANOVA> Data are presented as mean ± SEM. Significance was indicated as: * $p < 0.05$, ** $p < 0.01$, *** $p < 0.001$, not significant (ns).

Journal of Cell Science • Supplementary information

Supplemental figure 5

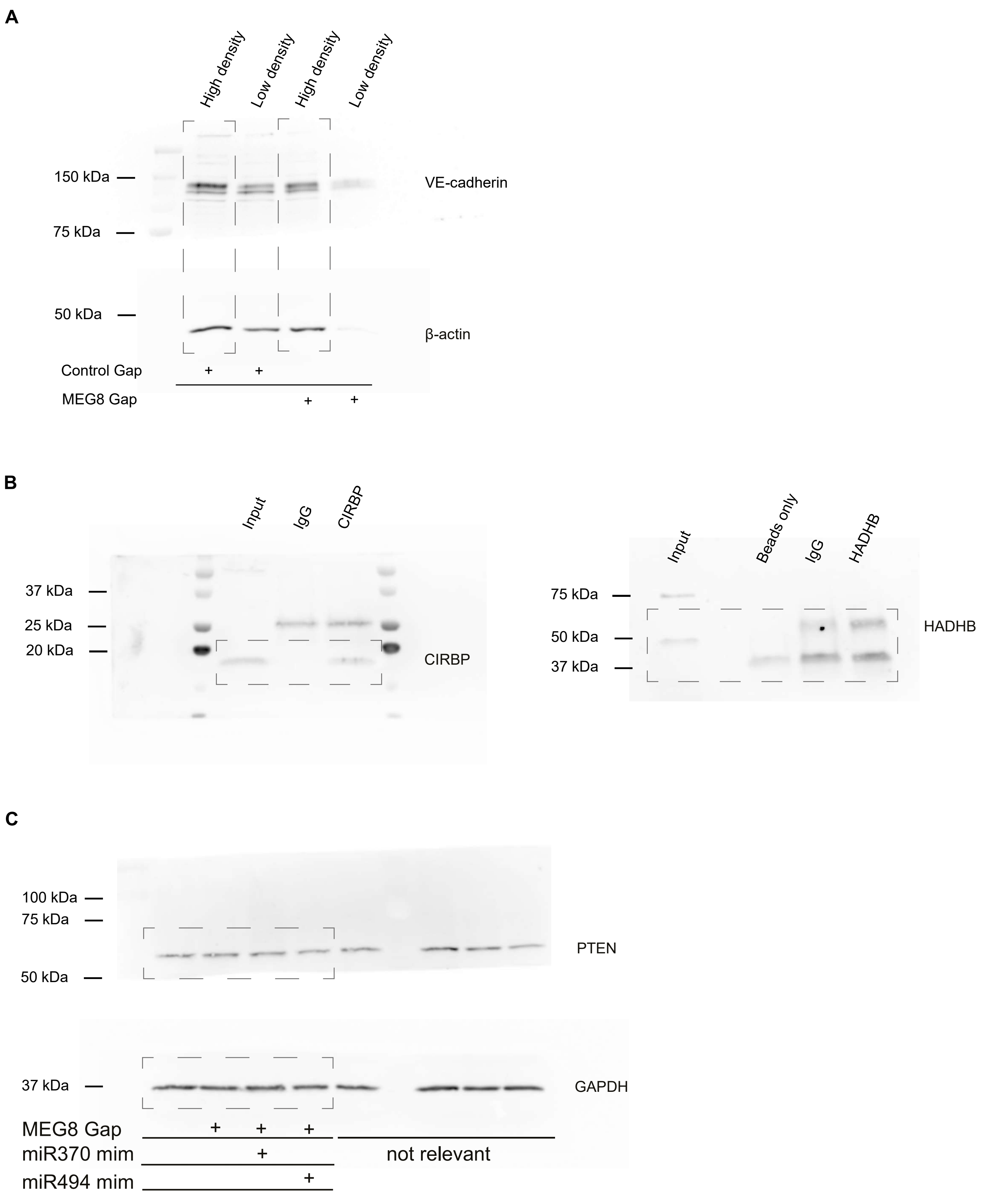

Fig. S5. Blot transparency. Full size original Western blots. **A**: Supplemental figure 2B. HUVECs were seeded at confluent (high density) or subconfluent (low density) densities. Cells seeded at high density were analysed to compare similar cell numbers. B: Supplemental figure 4B-C. Western blot following CLIP using CIRBP and HADHB antibody. For CIRBP, the IgG light chain is also visible at 25 kDa. For HADHB, the band which is visible at approximately 37 kDa is likely Protein G from the beads. **C**: Supplemental figure 5C.

Journal of Cell Science • Supplementary information

Table S1. RT-qPCR primers, oligos and antibodies used in this study.

Click here to download Table S1

Table S2. Analysis of RNA antisense purification and mass spectrometry.

Click here to download Table S2

Journal of Cell Science • Supplementary information
