# Supplementary material for: The long non-coding RNA MEG8 induces an endothelial barrier through regulation of microRNA-370 and -494 processing
Source: J Cell Sci. 2022 Jun 16;135(12):jcs259671. doi: 10.1242/jcs.259671 (PMC9270956; doi:10.1242/jcs.259671)
Supplement: Reviewer comments [file joces-135-259671_review_history.pdf]

The long non-coding RNA MEG8 induces an endothelial barrier through regulation of microRNA-370 and -494 processing

Veerle Kremer, Laura Stanicek, Eva van Ingen, Diewertje I. Bink, Sarah Hilderink, Anke J. Tijsen, Ilka Wittig, Lars Mäegdefessel, Anne Yaël Nossent and Reinier A. Boon
DOI: 10.1242/jcs.259671

Editor: Maria Carmo-Fonseca

Review timeline

Original submission:	6 December 2021
Editorial decision:	31 January 2022
First revision received:	19 April 2022
Accepted:	15 May 2022

Original submission

First decision letter

MS ID#: JOCES/2021/259671

MS TITLE: The long non-coding RNA MEG8 induces an endothelial barrier through regulation of microRNA-370 and -494 processing

AUTHORS: Veerle Kremer, Laura Stanicek, Eva van Ingen, Diewertje I. Bink, Sarah Hilderink, Anke J. Tijsen, Ilka Wittig, Lars Mäegdefessel, Anne Yaël Nossent, and Reinier A. Boon
ARTICLE TYPE: Research Article

We have now reached a decision on the above manuscript.

To see the reviewers' reports and a copy of this decision letter, please go to: https://submit-jcs.biologists.org and click on the 'Manuscripts with Decisions' queue in the Author Area. (Corresponding author only has access to reviews.)

As you will see, the reviewers raise a number of substantial criticisms that prevent me from accepting the paper at this stage. They suggest, however, that a revised version might prove acceptable, if you can address their concerns. If you think that you can deal satisfactorily with the criticisms on revision, I would be pleased to see a revised manuscript. We would then return it to the reviewers.

We are aware that you may be experiencing disruption to the normal running of your lab that makes experimental revisions challenging. If it would be helpful, we encourage you to contact us to discuss your revision in greater detail. Please send us a point-by-point response indicating where you are able to address concerns raised (either experimentally or by changes to the text) and where you will not be able to do so within the normal timeframe of a revision. We will then provide further guidance. Please also note that we are happy to extend revision timeframes as necessary.

Please ensure that you clearly highlight all changes made in the revised manuscript. Please avoid using 'Tracked changes' in Word files as these are lost in PDF conversion.

© 2022. Published by The Company of Biologists under the terms of the Creative Commons Attribution License (https://creativecommons.org/licenses/by/4.0/).

I should be grateful if you would also provide a point-by-point response detailing how you have dealt with the points raised by the reviewers in the 'Response to Reviewers' box. Please attend to all of the reviewers' comments. If you do not agree with any of their criticisms or suggestions please explain clearly why this is so.

Reviewer 1

Advance summary and potential significance to field

In this manuscript, Kremer et al. describe a role for the lncRNA MEG8 in regulating essential endothelial cell barrier function by regulating a subset of miRNAs present on the same genomic locus 14q32. The authors identified MEG8 as an upregulated lncRNA in high-passage HUVECs and its knockdown increased senescence and barrier loss. Mechanistically, MEG8 seems to regulate the processing of miRNA-370/-494 via the RNA-binding proteins CIRBP and HADHB.

Comments for the author

Altogether the manuscript provides interesting clues on the protective role of MEG8 in endothelial aging. However, the following concerns need to be addressed in order to provide careful and thoughtful pieces of evidence on the actual function of MEG8 in endothelial barrier function:

Major Comments:
1. In general, the authors should carefully reconsider their knockdown studies using ASO or siRNAs. Both knockdown strategies have been shown to cause multiple off-target effects also not connected to the actual target function. Therefore, the fact that the authors rely on only one ASO or one siRNA is not the best practice to draw robust conclusions. Therefore, I suggest that the authors perform the in vitro studies and miRNA expression determinations using at least two independent ASOs and siRNAs (three would be better) to clearly demonstrate that the knockdown of MEG8 is impairing the identified cellular functions. Moreover, the authors should consider including mismatched negative controls to further increase the robustness of their findings.
2. The cellular localization of lncRNAs is an important clue for their potential molecular function. How is the subcellular localization of MEG8 during aging? Is it predominantly nuclear as shown in a previous publication from the authors (Kremer et al., 2021)? The authors should perform subcellular localization followed by qPCR or single molecule RNA-FISH.
3. In Figure 2, the authors showed the role of MEG8 in endothelial barrier maintenance. Given the subsequent evidence on a post-transcriptional role of MEG8, is the ectopic overexpression of MEG8 capable of rescuing the barrier-loss phenotype?
4. In supplementary Figures 2E and F, the authors fail to show that the barrier function is impaired in high-passage HUVECs. This can be due to the difference in MEG8 expression between low and high passage HUVECs. How was the knockdown efficiency compared to low-passage cells? How was the expression level of residual MEG8 in high-passage cells compared to low-passage cells? The authors should check the knockdown efficiency as well as expression levels of MEG8 in knockdown samples between low and high passages. If higher, the residual MEG8 in high-passage cells might be enough to still be able to pursue its function. In the case of this scenario, please include these results as additional panels and comment/discuss them in the results/discussion sections. A potential way to solve this issue is to identify ASOs causing higher KD efficiency of MEG8 and perform the assay again.
5. In Figure 3B, the authors showed that MEG8 reduces the expression of selective miRNAs. The authors should invest more efforts in characterizing this regulatory axis. For instance, how are the targets of these miRNAs modulated once MEG8 is absent? Do the authors see the same effects on those targets upon miRNA knockdowns? Please provide pieces of evidence that MEG8 is regulating the functionality of its target miRNAs.
6. Are MEG8 and miRNA targets directly interacting through RNA-RNA interactions? Are binding sites for miRNA-370 and miRNA-494 in the sequence of MEG8? The authors should investigate the possibility of direct lncRNA-miRNA interaction between MEG8 and their miRNA targets. Is MEG8 acting as a sponge for the miRNA target functions?
7. Following comment 5. In Figure 4, the authors should also provide evidence that the mimic miRNAs are restoring the expression of targets regulated by miRNA-370 and miRNA-494.

© 2022. Published by The Company of Biologists under the terms of the Creative Commons Attribution License (https://creativecommons.org/licenses/by/4.0/).

Minor Comments:
1. Please improve the quality of the images in figure 1. From the provided images, it is not visible the difference presented in the quantification.
2. The authors should include every “data not shown” as supplementary figure panels.
3. In addition to major point 1, ASO knockdown approaches were considered to verify RNA transcript function over a transcription-based mechanism until two recent benchmark publications (see Lee et al. 2020, MolCell or Lai et al. 202, MolCell) showed that this is not valid anymore. Please add this point to authors’ discussion.
4. The authors should provide western blot images for CIRBP and HADHB CLIP experiments showing the enrichment for these two proteins. Moreover, for figures 3D and 3E, is there a positive control RNA target known to be bound by these factors?
5. Provide statistical significance also in figure 4A and B.

Reviewer 2

Advance summary and potential significance to field

The manuscript by Kremer and colleagues investigates the role of the long non-coding RNA MEG8 in endothelial cell barrier stability. It presents some interesting data in endothelial cell barrier function.

Comments for the author

There are a number of questions that need addressing. In particular the senescence link is not well developed.
1. In Fig 1A, expression of MEG8 in high passage EC looks like only 3/8 samples (assuming different isolates of HUVECs) are statistically increased. The variability is again seen in supplementary Figs1E-F where they analyse high passage cells. Hence it is not a robust observation. In Figure 1A are the 5 that are not changed non-senescent, while the 3 with high expression have become senescent? But if this is the case why does knock-down of MEG8 induce senescence? Does increase in expression levels as is seen in normal EC (supposedly as they age) actually inhibit proliferation? The knockdown for the rest of the experiments is of levels in normal proliferating young cells. SA beta gal is not enough to show that these are actually senescent. In all, the biology with MEG8 being protective in ageing cells and link to senescence, behind Figure 1 is confusing.
2. Is MEG8 regulated in other forms of senescence such as stress induced? This is rapid and independent of proliferation and maybe an easier system to address some of the questions in 1) above. It maybe particularly interesting to see if CIRBP is then regulated since it is stress responsive (line 297).
3. In the first paragraph of the Introduction the authors are talking about senescence and barrier function. Yet their system for Figures 2-4 are in 24 hour MEG8 depleted cells, which are unlikely to be senescent by that time frame. In addition, the cells are put at high density which again does not normally allow senescence to develop especially over the 24-48 hrs.
4. Figure 4A. The barrier function is not significant. But in Figure 2A it is. If the junctions are significantly decreased as demonstrated in Figure4B, why is the barrier function not significantly altered? It is not listed in the legends what all the dotted lines are-although assume repeats. Are they separate HUVEC isolates also?
5. The authors suggest that MEG8 is working through the miRNAs-370/494, as overexpression of these miRNAs rescues. However, they need to show that knockdown of these miRNAs recapitulates the MEG8 effects.
6. The authors spend some time identifying CIRBP and HADHB and their potential regulation of miR-370 and MiR-494. Are these proteins regulated on ageing? With senescence, proliferation etc?
7. The authors discuss MEG8 is reported to induce transcription of miR-34a (line 329). It is interesting that miR34 regulates senescence.

© 2022. Published by The Company of Biologists under the terms of the Creative Commons Attribution License (https://creativecommons.org/licenses/by/4.0/).

Reviewer 3

Advance summary and potential significance to field

In the manuscript “The long non-coding RNA MEG8 induces an endothelial barrier through regulation of microRNA-370 and -494 processing”, Kremer and colleagues demonstrate that MEG8 increases with passage number in HUVEC and iPSC derived human cardiomyocytes in culture, suggesting MEG8 is induced during aging. MEG8 knockdown resulted in reduced expression of miRNA-370 and -494 through interaction with CIRBP and HADHB. The data and analysis are potentially novel and interesting for the most part. The manuscript is clear and well written.

Comments for the author

One potential limitation is the usage of one single cell line for most of the experimental settings. It would be nice if some of the characterizations could be translated with the iPSC model. Otherwise, this model, and the corresponding figures, seem out of context. For instance, it will be interesting to see, if possible, microRNA-370 and -494 levels with cell passage.

Minor commentaries:
- The figures should clearly detail the number of independent samples.
- Line 137. I believe this is an overinterpretation regarding your datasets.
- Line 189. How was this miRNA subset selected?
- Line 282. Lack of reference.
- Line 313-316. This set of sentences are a bit confusing. Drosha is misspelled.

First revision

Author response to reviewers' comments

Reviewer 1 Advance Summary and Potential Significance to Field...

In this manuscript, Kremer et al. describe a role for the lncRNA MEG8 in regulating essential endothelial cell barrier function by regulating a subset of miRNAs present on the same genomic locus 14q32. The authors identified MEG8 as an upregulated lncRNA in high-passage HUVECs and its knockdown increased senescence and barrier loss. Mechanistically, MEG8 seems to regulate the processing of miRNA-370/-494 via the RNA-binding proteins CIRBP and HADHB.

Reviewer 1 Comments for the Author...

Altogether the manuscript provides interesting clues on the protective role of MEG8 in endothelial aging. However, the following concerns need to be addressed in order to provide careful and thoughtful pieces of evidence on the actual function of MEG8 in endothelial barrier function:

Major Comments:
1. In general, the authors should carefully reconsider their knockdown studies using ASO or siRNAs. Both knockdown strategies have been shown to cause multiple off-target effects also not connected to the actual target function. Therefore, the fact that the authors rely on only one ASO or one siRNA is not the best practice to draw robust conclusions. Therefore, I suggest that the authors perform the in vitro studies and miRNA expression determinations using at least two independent ASOs and siRNAs (three would be better) to clearly demonstrate that the knockdown of MEG8 is impairing the identified cellular functions. Moreover, the authors should consider including mismatched negative controls to further increase the robustness of their findings.

We thank the reviewers for taking time to review our manuscript. We are grateful for the suggestions, which are addressed below.

© 2022. Published by The Company of Biologists under the terms of the Creative Commons Attribution License (https://creativecommons.org/licenses/by/4.0/).

We have tested 3 additional GapmeRs, 2 of which showed effective knockdown of MEG8 (approximately 70-80%). Similar to our results with the GapmeR used throughout the manuscript, we observed no statistically significant changes in precursor miRNA levels. We did observe a significant decrease in mature miRNA levels, both miRNA-370 and -494. Similar to previous findings, we have measured endothelial barrier permeability and found an impaired barrier. Especially cell-cell contacts were reduced. We have tested 2 additional siRNAs, however the knockdown was not as effective. We observed approximately 50-60% knockdown for primer pair 2, and no statistically significant knockdown using primer 1. Primer pair 1 shows a similar trend, but there is a larger spread in the data. It should be noted that primer pair 1 targets upstream of the siRNA target site. It has been shown previously that siRNAs are less effective in targeting nuclear lncRNAs (Lennox et al. J of Rare Dis Res & Treatment 2016), which could explain these findings. Likely because of the incomplete knockdown, we did not observe a significant change in barrier function.

© 2022. Published by The Company of Biologists under the terms of the Creative Commons Attribution License (https://creativecommons.org/licenses/by/4.0/).

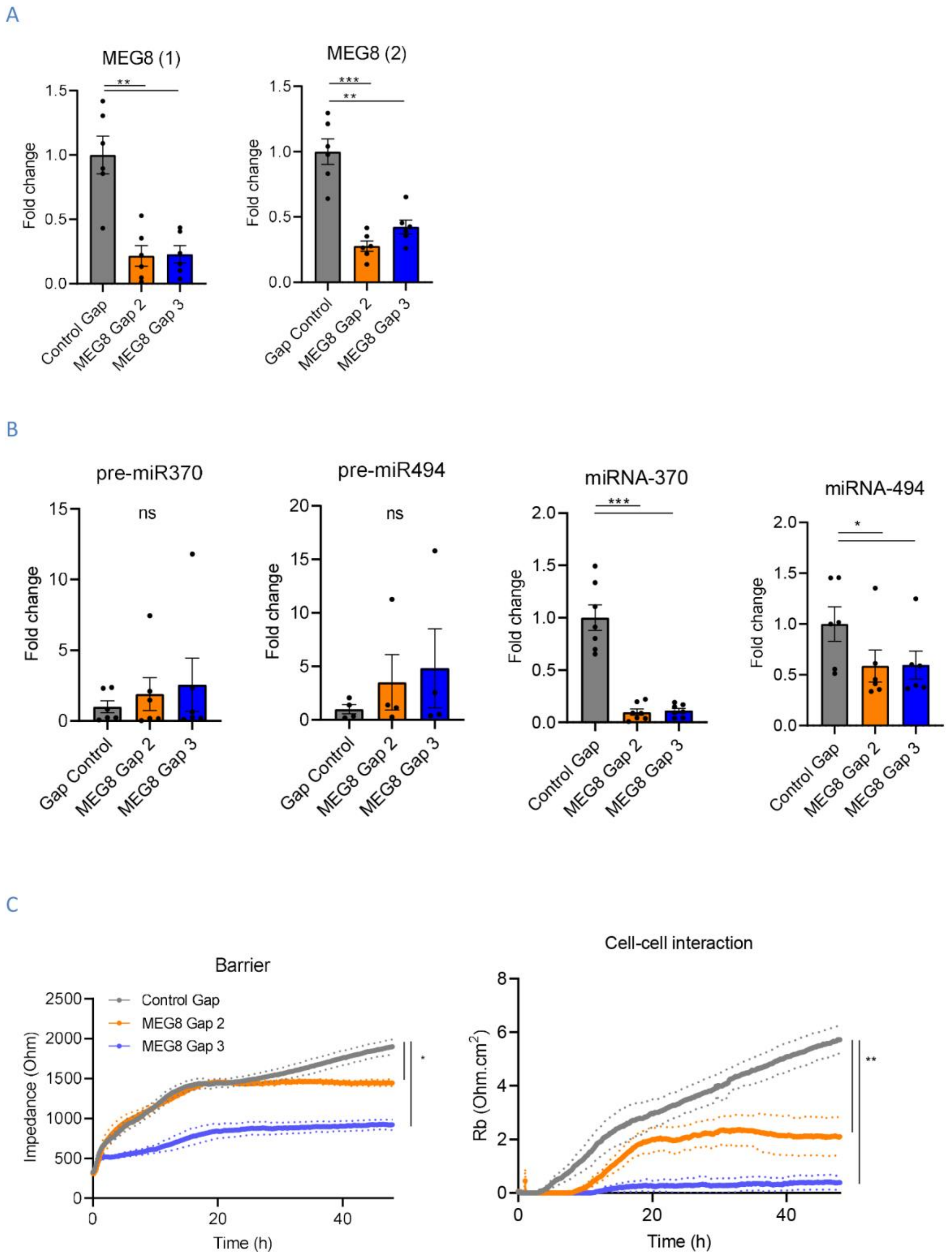

© 2022. Published by The Company of Biologists under the terms of the Creative Commons Attribution License (https://creativecommons.org/licenses/by/4.0/).

D

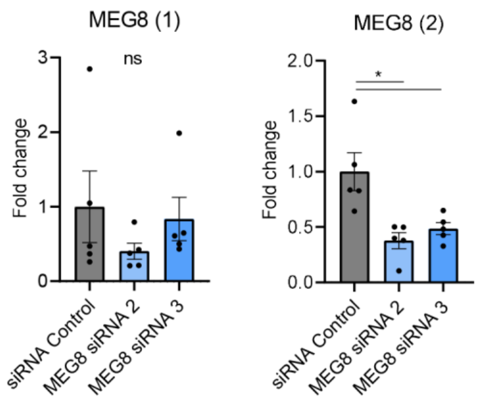

E

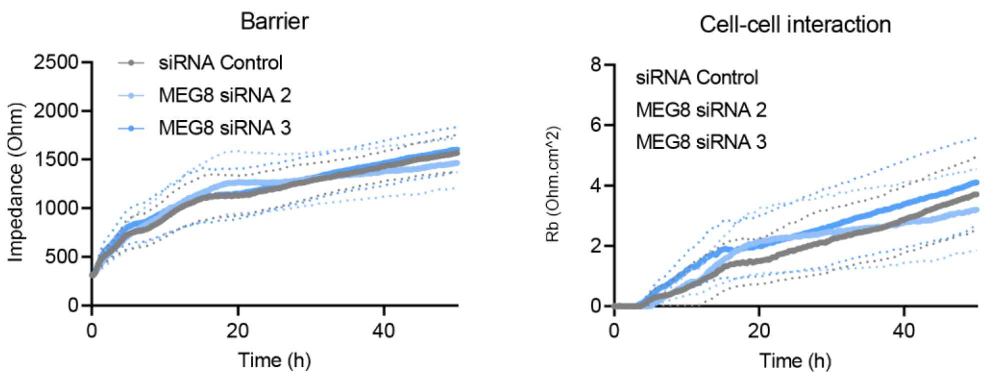

Reviewer figure 1: (A-B) HUVECs transfected using MEG8 targeting GapmeR and RNA was collected 48 hours after transfection. MEG8 expression was measured by RT-qPCR and normalized to RPLP0. For MEG8, 2 primer pairs were used. Primer pair 1 targets closest to the binding site of the Gapmer, primer pair 2 targets closest to the siRNA target site. (B) precursor miRNA expression was measured by RT-qPCR and normalized to U6. 6 experiments were performed. Mature miRNA expression was measured by RT-qPCR and normalized to miRNA-191. Here, data from 1 experiment were excluded because this was an outlier (measured using Graphpad). Groups were compared using paired ANOVA. 7 experiments were performed. (C) Seeding was done 24 hours after transfection at a density of 30 000 cells per well in 96W10E ECIS plates. Impedance was measured continuously. By altering the frequency, overall barrier and cell-cell contact can be distinguished over time.
Continuous lines indicate the mean, dotted lines indicate SEM. The area under the curve was quantified between 24 and 48 hours and groups were analysed using paired ANOVA. 4 experiments were performed. (D) HUVECs transfected using MEG8 targeting siRNA and RNA was collected 48 hours after transfection. MEG8 expression was measured by RT-qPCR and normalized to RPLP0. 5 experiments were performed. (E) Seeding was done 24 hours after transfection at a density of 30 000 cells per well in 96W10E ECIS plates. Impedance was measured continuously. By altering the frequency, overall barrier and cell-cell contact can be distinguished over time. Continuous lines indicate the mean, dotted lines indicate SEM. The area under the curve was quantified between 24 and 48 hours and groups were analysed using paired ANOVA. 3 experiments were performed. Data are presented as mean ± SEM. Significance was indicated as: * $p < 0.05$, **$p < 0.01$, ***$p < 0.001$.

2. The cellular localization of lncRNAs is an important clue for their potential molecular function. How is the subcellular localization of MEG8 during aging? Is it predominantly nuclear as shown in a previous publication from the authors (Kremer et al., 2021)? The authors should perform subcellular localization followed by qPCR or single molecule RNA-FISH.

We have repeated cellular fractionation experiments as described in the materials & methods using HUVECs at high passage. We observed predominantly nuclear localization of MEG8 in high

© 2022. Published by The Company of Biologists under the terms of the Creative Commons Attribution License (https://creativecommons.org/licenses/by/4.0/).

and low passage HUVECs.

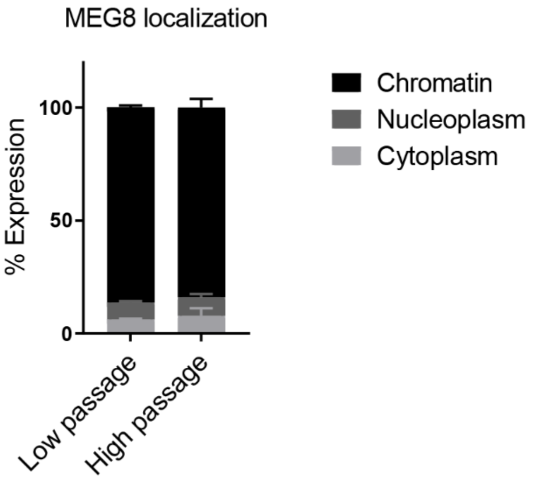

Reviewer figure 2: Cell fractionation following RT-qPCR in low (p2-4) and high (p13-14) passage HUVECs. RNA was extracted from the chromatin, nucleoplasm and cytoplasm of HUVECs. RT-qPCR was used to analyse MEG8 localization. Data are presented as mean ± SEM.

3. In Figure 2, the authors showed the role of MEG8 in endothelial barrier maintenance. Given the subsequent evidence on a post-transcriptional role of MEG8, is the ectopic overexpression of MEG8 capable of rescuing the barrier-loss phenotype?
This is indeed a very interesting suggestion. It should be noted that endothelial cells are rather difficult to transfect and therefore not ideal to perform overexpression. Within our department we regularly use lentiviral transduction, but this will not be suitable for MEG8 since this transcript is too large for this vector. The NCBI Reference Sequence NR_146000.1 is 4572 bp, and also does not include potentially alternatively spliced transcripts. Unfortunately we will not be able to perform overexpression experiments.

4. In supplementary Figures 2E and F, the authors fail to show that the barrier function is impaired in high-passage HUVECs. This can be due to the difference in MEG8 expression between low and high passage HUVECs. How was the knockdown efficiency compared to low-passage cells? How was the expression level of residual MEG8 in high-passage cells compared to low-passage cells? The authors should check the knockdown efficiency as well as expression levels of MEG8 in knockdown samples between low and high passages. If higher, the residual MEG8 in high-passage cells might be enough to still be able to pursue its function. In the case of this scenario, please include these results as additional panels and comment/discuss them in the results/discussion sections. A potential way to solve this issue is to identify ASOs causing higher KD efficiency of MEG8 and perform the assay again.

We have repeated the transfection in high passage cells and observed statistically significant knockdown of MEG8. In both low and high passage cells, we observed approximately 75% knockdown. This would suggest that knockdown is effective in both high and low passage cells. We did observe a trend towards further barrier impairment after MEG8 silencing in high passage cells.

We observed high variation in the different ECIS experiments, combined with the fact that we have fewer independent experiments and need to correct for multiple testing could explain why we do not observe statistically significant differences.

© 2022. Published by The Company of Biologists under the terms of the Creative Commons Attribution License (https://creativecommons.org/licenses/by/4.0/).

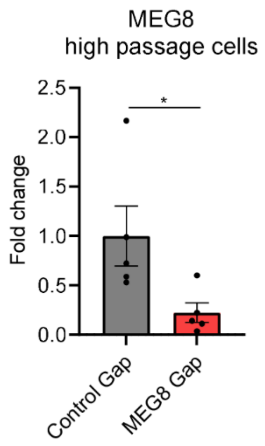

Reviewer figure 3: HUVECs (p13-15) were transfected with Control or MEG8 GapmeR. RNA was extracted after 48 hours and RT-qPCR was used to analyse MEG8 expression. Groups were compared using paired t-test. Data are presented as mean ± SEM. Significance was indicated as: * $p < 0.05$, **$p < 0.01$, ***$p < 0.001$, not significant (ns).

5. In Figure 3B, the authors showed that MEG8 reduces the expression of selective miRNAs. The authors should invest more efforts in characterizing this regulatory axis. For instance, how are the targets of these miRNAs modulated once MEG8 is absent? Do the authors see the same effects on those targets upon miRNA knockdowns? Please provide pieces of evidence that MEG8 is regulating the functionality of its target miRNAs.

We have selected PTEN as a potential downstream target since PTEN had been published previously to be a target of both miRNA-370 and miRNA-494 (Zeng et al. Journal of Oncology 2016; Yang et al. Oncology Reports 2015). Also, PTEN was thought to contribute to endothelial permeability (Kini et al. Journal Biol Chem 2010; Sanchez et al. Arterioscl, Throm, and Vasc Biol 2007). We have assessed PTEN protein levels after MEG8 knockdown and miRNA overexpression. We observed an increase in PTEN protein after MEG8 knockdown which was restored by miRNA-494 overexpression, although this effect was not statistically significant. Functionally, silencing of PTEN indeed restored EC barrier function after MEG8 knockdown, similar to miRNA overexpression. To further assess the effect of MEG8 on predicted miRNA targets, we selected proteins which were predicted to be targeted by both miRNA-370 and -494. The following databases were used: miRWalk, miRanda, RNA22 and TargetScan. We obtained mRNA expression levels after MEG8 knockdown. The dataset can be found at GSE186616. When comparing the fold change of predicted miRNA targets to all genes regulated by MEG8, we observed a shift towards increased expression of miRNA targets after loss of MEG8 (Figure 4E). This would suggest that loss of MEG8 and subsequent reduction in miRNA expression results in a trend towards increased expression of miRNA target genes. It should be noted that not all genes were significantly altered by MEG8 knockdown. Taken together, these results suggest MEG8 contributes to endothelial barrier maintenance through regulation of specific miRNA expression.

© 2022. Published by The Company of Biologists under the terms of the Creative Commons Attribution License (https://creativecommons.org/licenses/by/4.0/).

A

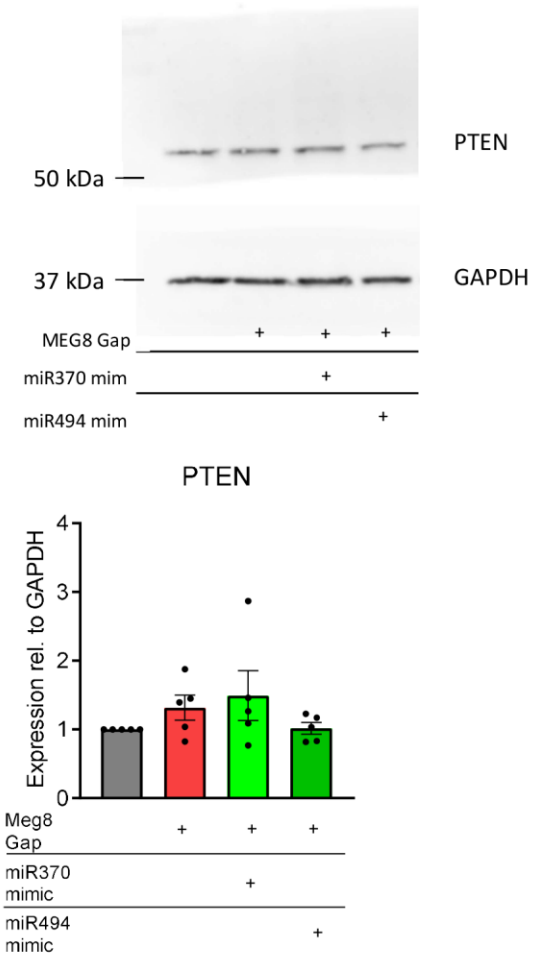

B

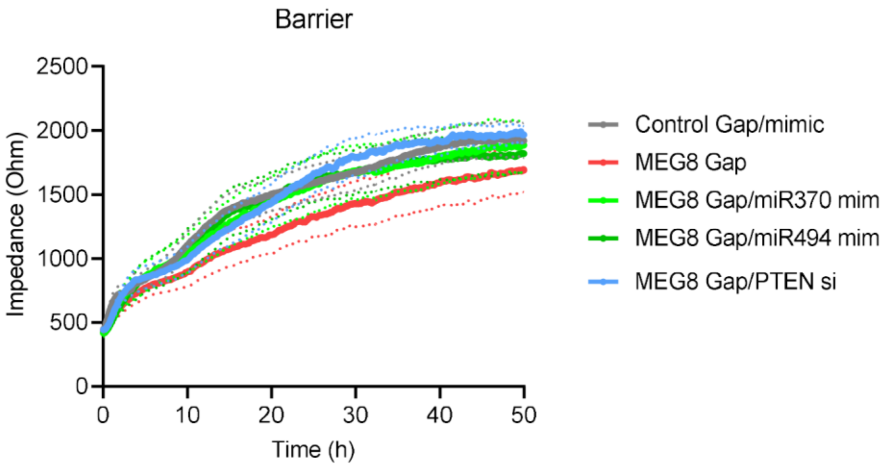

© 2022. Published by The Company of Biologists under the terms of the Creative Commons Attribution License (https://creativecommons.org/licenses/by/4.0/).

C

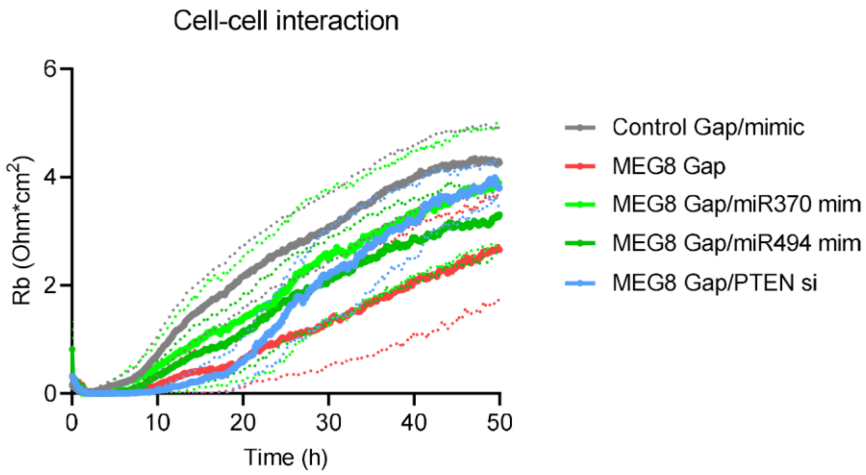

D

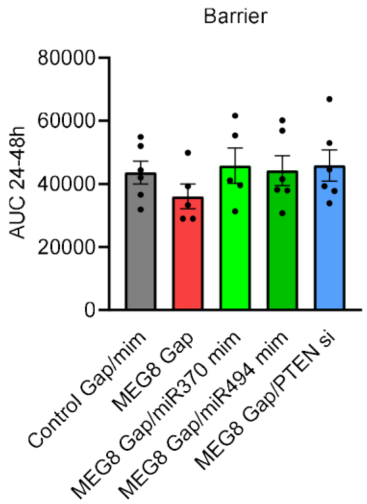

E

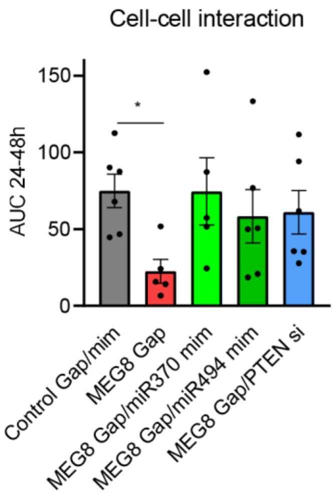

F

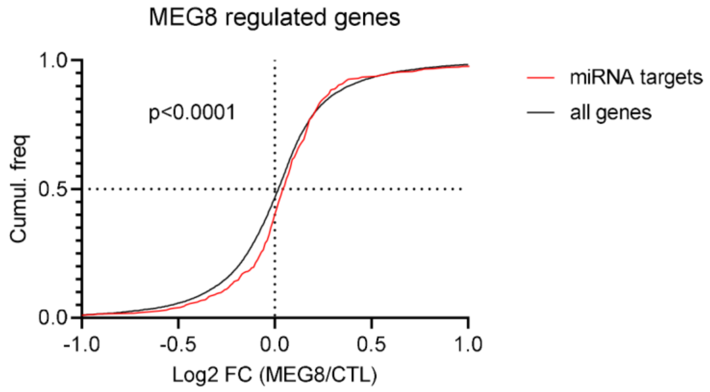

Reviewer figure 4: (A) PTEN protein levels were determined using Western blot. Cell lysates were collected 48 hours after transfection. GAPDH was used as a loading control. Images were cropped for clarity. Band intensity was quantified using ImageQuant. (B-C) HUVECs transfected and seeded 24 hours after transfection at a density of 100 000 cells per well in 8W10E ECIS plates. Impedance was measured continuously. By altering the frequency, overall barrier (B) and cell-cell contact (C) can be distinguished over time. Continuous lines indicate

© 2022. Published by The Company of Biologists under the terms of the Creative Commons Attribution License (https://creativecommons.org/licenses/by/4.0/).

the mean, dotted lines indicate SEM. (D-E): The area under the curve of (B) and (C) was quantified between 24 and 48 hours and groups were analysed using paired ANOVA. (F) Gene expression in Control and MEG8 GapmeR 1 treated HUVECs was obtained from GSE186616. miRNA targets were predicted using online available tools miRWalk, miRanda, RNA22 and TargetScan. The FC of miRNA targets was compared to all genes regulated by MEG8 knockdown. Groups were compared using a Kolmogorov-Smirnov test. Data are presented as mean ± SEM. Significance was indicated as: * $p < 0.05$, **$p < 0.01$, ***$p < 0.001$, not significant (ns).

6. Are MEG8 and miRNA targets directly interacting through RNA-RNA interactions? Are binding sites for miRNA-370 and miRNA-494 in the sequence of MEG8? The authors should investigate the possibility of direct lncRNA-miRNA interaction between MEG8 and their miRNA targets. Is MEG8 acting as a sponge for the miRNA target functions?

To investigate potential interaction of miRNA-370/-494 and MEG8, we have used pulldown as described previously in the manuscript. We have measured miRNA expression in the input and elution fractions. We were not able to detect expression of miRNA-370 in the elution fraction of either scrambled or MEG8 oligo samples. We were able to detect miRNA-494 enrichment in MEG8 oligo elution fractions compared to scrambled control, although enrichment varied between experiments. It should be noted that we cannot exclude that MEG8 and miRNA-494 are a part of a larger complex and are not binding directly. Inhibition of miRNAs did not affect expression of MEG8, which would argue against miRNA binding to MEG8 and inhibiting its expression. We also investigated potential miRNA-370/-494 binding sites in MEG8 using online available tools such as Starbase, DIANA and miRNET but we did not find any potential binding sites using these tools. The fact that miRNAs are downregulated upon MEG8 knockdown argues against the idea that MEG8 acts as a miRNA sponge. In that case, we would expect an upregulation of miRNAs after MEG8 inhibition.

A

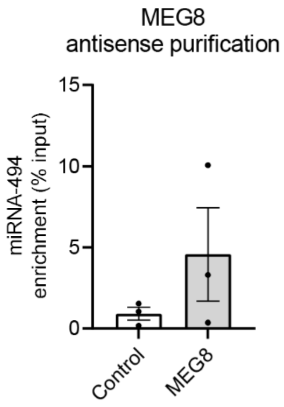

B

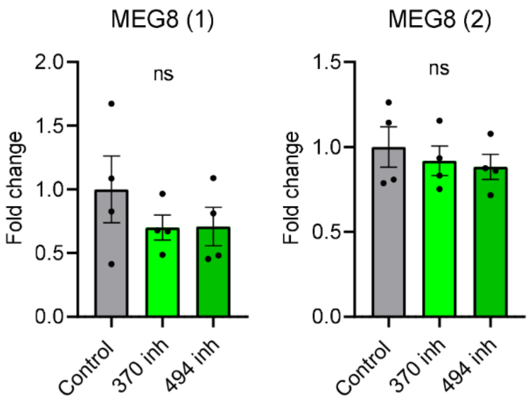

© 2022. Published by The Company of Biologists under the terms of the Creative Commons Attribution License (https://creativecommons.org/licenses/by/4.0/).

Reviewer figure 5: (A) Elutions from MEG8-antisense purification were analysed by RT-qPCR. miRNA- 370 was not detected and miRNA-494 enrichment is shown as a percentage of the input. 3 experiments were performed. (B) miRNA inhibitors were transfected and RNA was collected after 48 hours. MEG8 expression was measured by RT-qPCR using primer pairs 1 and 2. Groups were compared using paired ANOVA. 5 experiments were performed. Data are presented as mean ± SEM. Significance was indicated as: * $p < 0.05$, **$p < 0.01$, ***$p < 0.001$, not significant (ns).

7. Following comment 5. In Figure 4, the authors should also provide evidence that the mimic miRNAs are restoring the expression of targets regulated by miRNA-370 and miRNA-494.

As explained above (point 5), overexpression of miR-494 mimics, but not miR-370 mimics, restored the expression of PTEN after knockdown of MEG8.

Minor Comments:
1. Please improve the quality of the images in figure 1. From the provided images, it is not visible the difference presented in the quantification.
We have included zoomed-in pictures for clarification.

2. The authors should include every “data not shown” as supplementary figure panels.
We have included the RNase H accessibility assay (Supplementary figure 3A) which was used to select a suitable oligo for affinity pulldown. Probe 4 showed the lowest MEG8 expression, indicating that this region is accessible for degradation by RNase H.

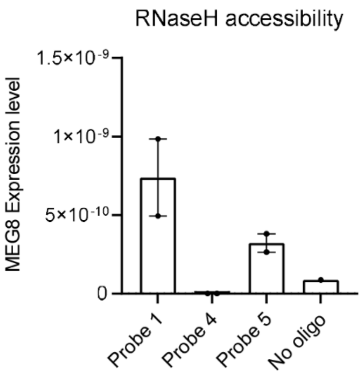

Reviewer figure 6: DNA oligonucleotides targeting the RNA of interest were designed to analyse the accessibility of multiple sections of the RNA. Cell lysate was incubated with DNA oligonucleotide and treated with RNase H. Primers were designed to amplify by RT-qPCR a region of approximately 150 nucleotides around the potentially bound DNA oligonucleotide. 2 experiments were performed. Data are presented as mean ± SEM.

3. In addition to major point 1, ASO knockdown approaches were considered to verify RNA transcript function over a transcription-based mechanism until two recent benchmark publications (see Lee et al. 2020, MolCell or Lai et al. 202, MolCell) showed that this is not valid anymore. Please add this point to authors’ discussion.

We have included this in the discussion.

4. The authors should provide western blot images for CIRBP and HADHB CLIP experiments showing the enrichment for these two proteins. Moreover, for figures 3D and 3E, is there a positive control RNA target known to be bound by these factors?

We have found 1 publication where HADHB was shown to bind Renin (REN)-3’UTR (Adams et al. JBC 2003). However, the primers form the publication show high background and are not suitable to use as a positive control RNA target. According to data from the Human Protein Atlas, REN mRNA expression is very low in endothelial cells. Therefore we do not think we can design other primers to quantify REN enrichment to HADHB.
CIRBP was shown to bind SSR2 (Liu et al. Sci Rep 2013; Indacochea RNA 2021). SSR2 was

© 2022. Published by The Company of Biologists under the terms of the Creative Commons Attribution License (https://creativecommons.org/licenses/by/4.0/).

measured as a positive control RNA target, and indeed we observed enrichment of SSR2 in the CIRBP fraction compared to IgG.

To assess CIRBP and HADHB enrichment by Western blot, we used the CLIP protocol as described in the materials & methods. We observed both CIRBP and HADHB enrichment in the antibody-of- interest fraction compared to IgG. We made one adjustment to the protocol, for the HADHB IP and Western blot we did not perform crosslinking, since previous blots had shown high background signal.

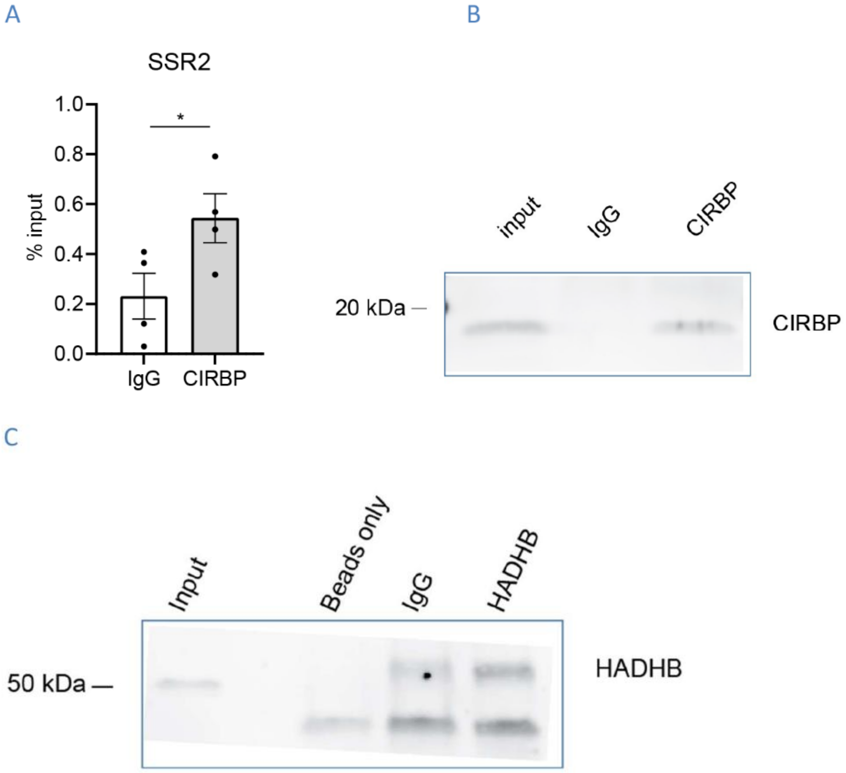

Reviewer figure 7: (A) SSR2 binding to CIRBP analysed in HUVECs by RT-qPCR following CLIP. Non- targeting IgG was used as a control. Enrichment was quantified relative to input. 4 individual experiments were performed. Groups were compared using a paired t-test. (B) CIRBP enrichment was assessed by Western blot following CLIP. Input is approximately 2% of the total lysate. The predicted size of CIRBP is 19 kDa. The IgG light chain is also visible at 25 kDa (supplemental figure 4B). (C) HADHB enrichment was assessed by Western blot following CLIP. Input is approximately 2% of the total lysate. The predicted size of HADHB is 50 kDa. The band which is visible at approximately 37 kDa is likely Protein G from the beads.

5. Provide statistical significance also in figure 4A and B.
Since the lines are quite close together, indicating significance within the graph is not clear. The bar graphs below correspond to the ECIS data, and these include indications of statistical significance. We have clarified this in the legend.

Reviewer 2 Advance Summary and Potential Significance to Field...

The manuscript by Kremer and colleagues investigates the role of the long non- coding RNA MEG8 in endothelial cell barrier stability. It presents some interesting data in endothelial cell barrier function.

Reviewer 2 Comments for the Author...

There are a number of questions that need addressing. In particular the senescence link is not well developed.

© 2022. Published by The Company of Biologists under the terms of the Creative Commons Attribution License (https://creativecommons.org/licenses/by/4.0/).

1. In Fig 1A, expression of MEG8 in high passage EC looks like only 3/8 samples (assuming different isolates of HUVECs) are statistically increased. The variability is again seen in supplementary Figs1E-F where they analyse high passage cells. Hence it is not a robust observation. In Figure 1A are the 5 that are not changed non-senescent, while the 3 with high expression have become senescent? But if this is the case why does knock-down of MEG8 induce senescence?
Does increase in expression levels as is seen in normal EC (supposedly as they age) actually inhibit proliferation? The knockdown for the rest of the experiments is of levels in normal proliferating young cells. SA beta gal is not enough to show that these are actually senescent. In all, the biology with MEG8 being protective in ageing cells and link to senescence, behind Figure 1 is confusing.

We thank the reviewer for their suggestions, which are addressed below.

The samples in Figure 1A are not matched, so we have used an unpaired test. Since there was a large spread between the samples, we used a Mann Whitney test to compare the rank rather than the mean. We have included a violin plot of the same data of Figure 1A to better show that MEG8 expression is higher on average in high passage cells.

To further clarify whether cells undergo senescence after loss of MEG8, we have measured additional markers of senescence as described by González-Gualda et al. (FEBS Journal, 2021). We observed a significant increase in p21 mRNA expression and a trend towards increased IL-1a and MMP1 expression. IL-6 and p16 were not changed. Also, proliferation was decreased. These results would suggest that cells are showing hallmarks of senescence upon MEG8 depletion, although the cells are likely not fully senescent.

Regarding the HUVECs, they are not separate isolates. We obtained commercially available cells from Lonza. We have used 2 different batches of cells (#1028 and #1032), each are a mix of 5 different donors. Independent experiments were performed over several weeks and at different passages.

A

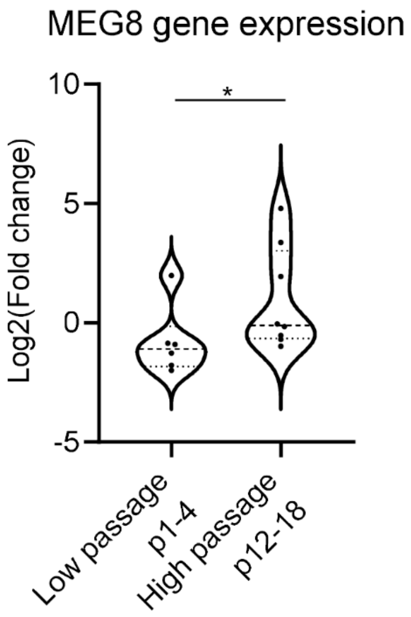

© 2022. Published by The Company of Biologists under the terms of the Creative Commons Attribution License (https://creativecommons.org/licenses/by/4.0/).

B

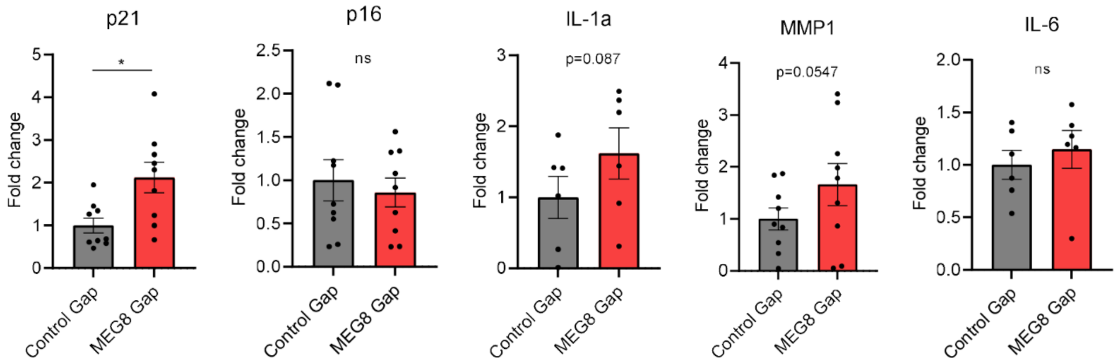

C

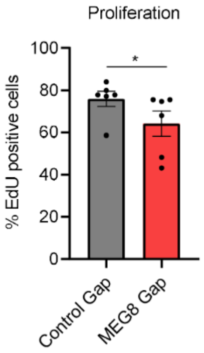

Reviewer figure 8: (A) MEG8 expression was measured by RT-qPCR in HUVECs at low and high passage. Low passage cells were between passage 1 and 4. High passage cells were between passage 12 and 18. Expression was normalized to RPLP0 and log transformed. Groups were compared using Mann-Whitney test. (B) HUVECs were transfected with MEG8 or Control GapmeR and gene expression levels were measured 48 hours after transfection by RT-qPCR. Expression is relative to RPLP0. Groups were compared using paired t-test. (C) Proliferation was measured by EdU incorporation between 24-48 hours after transfection. The Click-iT EdU proliferation kit (Thermo Fisher) was used according to the manufacturers' instructions. The percentage of proliferating cells is shown. Groups were compared using a paired t-test. Data are presented as mean ± SEM.

2. Is MEG8 regulated in other forms of senescence such as stress induced? This is rapid and independent of proliferation and maybe an easier system to address some of the questions in 1) above. It may be particularly interesting to see if CIRBP is then regulated since it is stress responsive (line 297).

Casella et al. (Nucleic Acids Research, 2019) observed a 2-fold induction in MEG8 expression in Human Aortic Endothelial Cells (HAEC) after irradiation-induced senescence. The authors observed no change in CIRBP or HADHB expression in the same cells. We exposed HUVECs to doxorubicin to induce DNA damage and cell stress. We observed an induction of p21 expression, suggesting cells are less proliferative. We observed no change in MEG8, CIRBP or HADHB RNA expression. We also exposed HUVECs to hypoxia. We observed a trend towards reduced CIRBP upon hypoxia exposure, but observed no statistically significant changes in CIRBP or HADHB expression. This could be due to the time of exposure or the oxygen level.

© 2022. Published by The Company of Biologists under the terms of the Creative Commons Attribution License (https://creativecommons.org/licenses/by/4.0/).

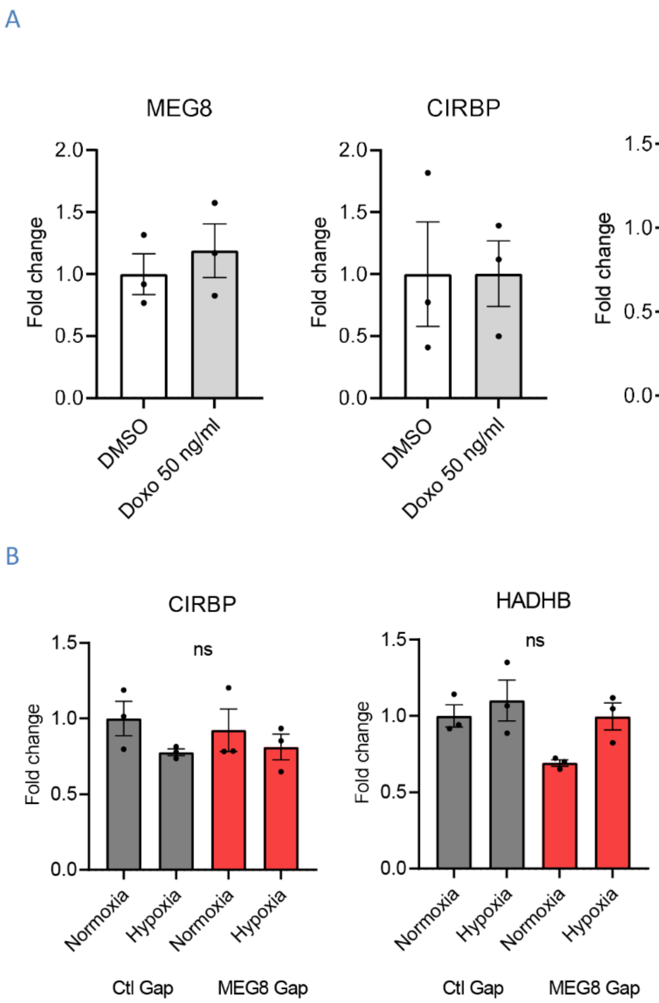

Reviewer figure 9: (A) HUVECs were exposed to doxorubicin (50 ng/ml) or DMSO control for 48 hours. Medium was replaced with full ECM for an additional 24 hours. RNA was isolated and gene expression was measured by RT-qPCR and normalized to RPLP0. Groups were compared using a t- test. (B) HUVECs were transfected with Control or MEG8 GapmeR. After 24 hours, cells were exposed to 1 % hypoxia or normoxia for 24 hours and RNA was collected. Expression was normalized to RPLP0. Groups were compared using ANOVA. Data are presented as mean ± SEM.

3. In the first paragraph of the Introduction the authors are talking about senescence and barrier function. Yet their system for Figures 2-4 are in 24 hour MEG8 depleted cells, which are unlikely to be senescent by that time frame. In addition, the cells are put at high density which again does not normally allow senescence to develop especially over the 24-48 hrs.

We have further addressed the development of senescence in question 1. Based on these findings we agree that cells likely are not fully senescent, but are showing some of the hallmarks such as reduced proliferation and an increase in senescence-associated β-galactosidase.

4. Figure 4A. The barrier function is not significant. But in Figure 2A it is. If the junctions are significantly decreased as demonstrated in Figure4B, why is the barrier function not significantly altered? It is not listed in the legends what all the dotted lines are-although assume repeats. Are they separate HUVEC isolates also?

The trend is similar in Figure 4A, although not statistically significant. This could be in part due to a larger variation and correcting for multiple testing. Furthermore, for this experiment cells are transfected with both a GapmeR and miRNA mimic, which means that the total amount of transfected oligo is 100 µM instead of 50 µM.

© 2022. Published by The Company of Biologists under the terms of the Creative Commons Attribution License (https://creativecommons.org/licenses/by/4.0/).

The continuous lines indicate the mean, the dotted lines indicate SEM. We have clarified this in the figure legend.

5. The authors suggest that MEG8 is working through the miRNAs-370/494, as overexpression of these miRNAs rescues. However, they need to show that knockdown of these miRNAs recapitulates the MEG8 effects.
We have repeated ECIS experiments using miRNA inhibitors of miRNA-370 and miRNA-494. We observed a decrease in overall barrier function as well as a decrease in cell-cell contacts upon miRNA inhibition. The effect of miRNA inhibitors was not as pronounced as knockdown of MEG8. This would suggest that MEG8 could have additional effects which could affect the endothelial barrier independent of miRNA-370/-494 expression.

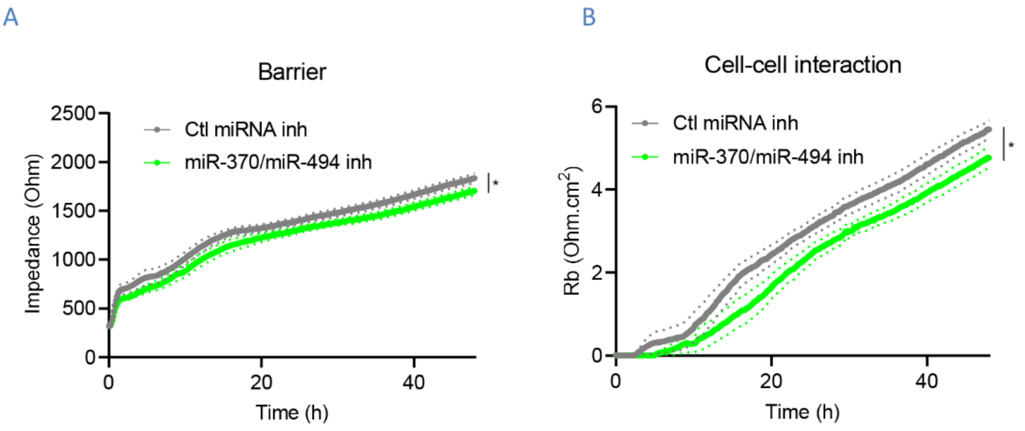

Reviewer figure 10: HUVECs transfected and seeded 24 hours after transfection at a density of 30 000 cells per well in 96W10E ECIS plates. Impedance was measured continuously. By altering the frequency, overall barrier (A) and cell-cell contact (B) can be distinguished over time. Continuous lines indicate the mean, dotted lines indicate SEM. The area under the curve of (A) and (B) was quantified between 24 and 48 hours and groups were analysed using paired t-test. In total, 7 experiments were performed. Data are presented as mean ± SEM. Significance was indicated as: * $p < 0.05$, ** $p < 0.01$, *** $p < 0.001$, not significant (ns).

6. The authors spend some time identifying CIRBP and HADHB and their potential regulation of miR-370 and MiR-494. Are these proteins regulated on ageing? With senescence, proliferation etc?
We did not observe changes in CIRBP/HADHB mRNA expression after loss of MEG8 or at high passage. Also, we did not observe a change in expression levels after exposure to doxorubicin (question 2). We do not nececarily expect these proteins to be affected at the RNA level by MEG8. Our data indicates an interaction between MEG8 and CIRBP/HADHB protein, and we hypothesize that MEG8 is required for the formation of a complex rather than regulation of expression levels.

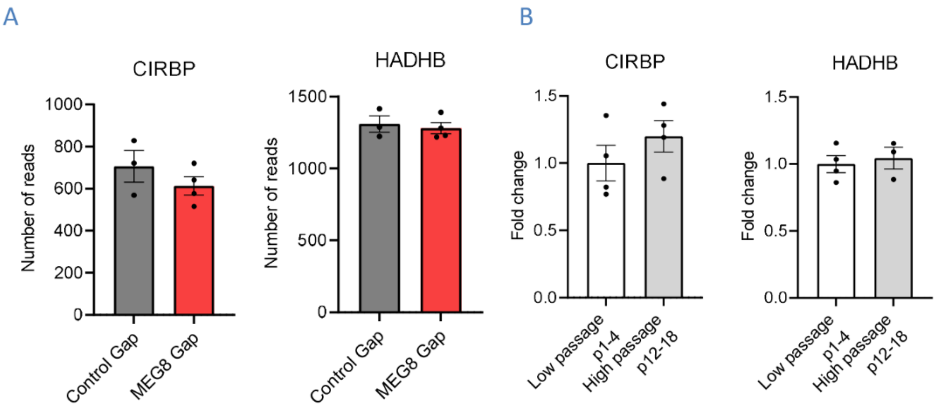

© 2022. Published by The Company of Biologists under the terms of the Creative Commons Attribution License (https://creativecommons.org/licenses/by/4.0/).

Reviewer figure 11: (A) Changes in gene expression after GapmeR-mediated silencing of MEG8 were analysed by RNA sequencing as described previously (Kremer et al., Sci Rep 2022). RNA was collected 48 hours after transfection. (B) Gene expression was measured by RT-qPCR in HUVECs at low and high passage. Low passage cells were between passage 1 and 4. High passage cells were between passage 12 and 18. Expression was normalized to RPLP0. Data are presented as mean ± SEM.

7. The authors discuss MEG8 is reported to induce transcription of miR-34a (line 329). It is interesting that miR34 regulates senescence.
Terashima et al. observed transcriptional repression of miRNA-34a after MEG8 overexpression. The finding that miR34a regulates senescence is indeed interesting, and these two observations would indeed be consistent with a more senescent phenotype upon MEG8 depletion. We have included this in the discussion section.

Reviewer 3 Advance Summary and Potential Significance to Field...

In the manuscript "Long non-coding RNA MEG8 induces endothelial barrier through regulation of microRNA-370 and -494 processing", Kremer and colleagues demonstrate that MEG8 increases with passage number in HUVEC and iPSC derived human cardiomyocytes in culture, suggesting MEG8 is induced during aging.
MEG8 knockdown resulted in reduced expression of miRNA-370 and -494 through interaction with CIRBP and HADHB. The data and analysis are potentially novel and interesting for the most part. The manuscript is clear and well written.

Reviewer 3 Comments for the Author...

One potential limitation is the usage of one single cell line for most of the experimental settings. It would be nice if some of the characterizations could be translated with the iPSC model. Otherwise, this model, and the corresponding figures, seem out of context. For instance, it will be interesting to see, if possible, microRNA-370 and -494 levels with cell passage.

We thank the reviewer for their suggestions, which are addressed below.

Regarding the cells, they are primary cells rather than a cell line. As stated previously, the HUVECs are not separate isolates. Instead, we have used 2 batches of cells from Lonza and performed independent experiments at different weeks. Each batch contained cells from 5 separate donors, both male and female.

To further characterize the iPSC cardiomyocytes, we measured miRNA expression in the same iPSC samples. miRNA-370 was not detectable in these cells. Similar to the findings regarding MEG8 expression, we observed an increase in miRNA-494 expression at high passage, although this was not statistically significant.

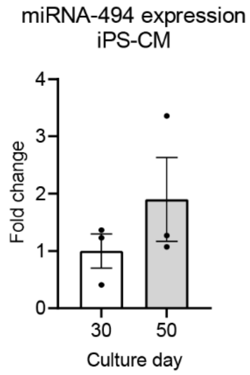

© 2022. Published by The Company of Biologists under the terms of the Creative Commons Attribution License (https://creativecommons.org/licenses/by/4.0/).

Reviewer figure 12: miRNA expression was measured by RT-qPCR in iPSC derived cardiomyocytes. Cells cultured for 30 days were compared to 50 days in culture. Expression was normalized to miRNA-191. Data are presented as mean ± SEM.

Minor commentaries:

- The figures should clearly detail the number of independent samples.

We have included the number of individual experiments in the figure legends.

- Line 137. I believe this is an overinterpretation regarding your datasets.

We have changed the title of the section to: MEG8 expression is increased in high passage HUVECs.

- Line 189. How was this miRNA subset selected?

  We have clarified the selection in the results section.

- Line 282. Lack of reference.

  We have included the reference.

- Line 313-316. This set of sentences are a bit confusing. Drosha is misspelled.

  We have clarified the text and corrected any spelling errors.

Second decision letter

MS ID#: JOCES/2021/259671

MS TITLE: The long non-coding RNA MEG8 induces an endothelial barrier through regulation of microRNA-370 and -494 processing

AUTHORS: Veerle Kremer, Laura Stanicek, Eva van Ingen, Diewertje I. Bink, Sarah Hilderink, Anke J. Tijsen, Ilka Wittig, Lars Mäegdefessel, Anne Yaël Nossent and Reinier A. Boon
ARTICLE TYPE: Research Article

I am happy to tell you that your manuscript has been accepted for publication in Journal of Cell Science, pending standard ethics checks.

Reviewer 1

Advance summary and potential significance to field

First of all, I would like to thank the authors for the extra care in addressing my comments. Overall, the authors responded adequately to all my suggestions and added very important data, significantly improving the manuscript. I also think that they have responded adequately to the other reviewers' concerns.

I take this opportunity to wish them all the best of luck in their future career paths.

Comments for the author

I do not have any additional comments. For this reason, I fully support the publication of this revised manuscript in the Journal of Cell Science.

© 2022. Published by The Company of Biologists under the terms of the Creative Commons Attribution License (https://creativecommons.org/licenses/by/4.0/).
